# Generation of a high-intensity, superthermal muonium beam for gravity and laser spectroscopy experiments

**DOI:** 10.1038/s41567-026-03433-x

**Published:** 2026-09-14

**Authors:** J. Zhang, A. Antognini, M. Bartkowiak, D. Goeldi, K. Kirch, A. Knecht, D. Taqqu, R. Waddy, F. Wauters, P. Wegmann, A. Soter

**Affiliations:** 1 Institute for Particle Physics and Astrophysics, ETH Zürich, Zürich, Switzerland; 2 PSI Center for Neutron and Muon Sciences, Villigen-PSI, Switzerland; 3 Institute for Nuclear Physics, Johannes Gutenberg-University, Mainz, Germany

**Keywords:** Experimental particle physics, Matter waves and particle beams, Exotic atoms and molecules

## Abstract

The universality of free fall, a cornerstone of Einstein’s theory of gravity, has so far only been tested with neutral composite states of first-generation standard model particles, such as atoms, neutrons and antihydrogen. Muonium, the bound state of a positively charged anti-muon and an electron, offers the possibility to probe gravity in the absence of the strong interaction with second-generation standard model particles. However, the short muon lifetime and the existing diffuse thermal muonium sources rendered such measurements unfeasible. Here we report the generation of a high-brightness muonium beam, which we extract from a thin layer of superfluid helium. The mean longitudinal velocity and narrow spread of the velocity distribution indicate a superthermal beam, and yields are similar to the highest-intensity diffuse sources. This beam is expected to enable muonium interferometry and a per-cent-level measurement of its gravitational acceleration. In addition, its unprecedented brightness opens the way to sub-kilohertz 1S–2S spectroscopy, enabling the precise determination of the muon mass and stringent tests of bound-state quantum electrodynamics.

## Abstract (web summary)

A high-intensity, superthermal beam is generated by extracting muonium from a thin layer of superfluid helium. This beam can be used for tests of gravity and laser spectroscopy.

## Main

Muonium (Mu) is a hydrogen-like exotic atom, where a positive anti-muon (μ^+^) and an electron (e^−^) are bound by the Coulomb interaction. This purely leptonic system is a unique precision probe to test bound-state quantum electrodynamics without the influence of nuclear and finite-size effects^1^. Laser spectroscopy of the Mu 1S–2S transition^2^, and microwave spectroscopy of the Mu ground-state hyperfine structure^3^ provide precision measurements of fundamental constants like the muon mass and magnetic moment, whereas searches for Mu–anti-Mu conversion constrain charged lepton number violation^4^. Future studies and presently ongoing efforts for improvements^5–7^ are strongly motivated by other precision measurements like the anomalous magnetic moment of the muon ^8,9^, and the theoretical possibilities for finding new physics^10–12^.

Another unique and so far unexplored facet of Mu is that its mass is dominated by the μ^+^ mass, which is not only an elementary antiparticle but also a second-generation lepton. Direct measurements of its gravitational acceleration (*g*_Mu_) would provide a unique way to test the universality of free fall and, hence, Einstein’s weak equivalence principle^13^ in higher generations of the standard model. The weak equivalence principle was rigorously tested using normal matter probes in laboratory-based^14^ and satellite-borne^15^ experiments to ~10^−15^ fractional precision. Less-precise studies extended the mass scale from single atoms^16,17^ and neutrons^18^ to celestial objects exerting their own gravitational field^19^. Since the relative strength of gravity is ~30–40 orders of magnitude weaker than the electromagnetic interaction between charged particles, neutral exotic atoms are so far the only feasible platforms to directly study gravity in other sectors of the standard model. Recently, the ALPHA experiment at CERN confirmed that antihydrogen ($\bar{{\rm{H}}}=\bar{\rm{p}}+{\rm{e}}^{+}$) falls towards Earth with an acceleration consistent with normal matter^20^. Earlier indirect measurements using charged particles in Penning traps^21,22^, and neutral meson oscillations^23,24^ also implied equivalency between matter and antimatter. Further experiments using antihydrogen^25,26^ and positronium (Ps = e^−^ + e^+^) (ref. ^27^) have been proposed.

However, all gravitational free-fall measurements so far involved composite hadrons, namely, protons, neutrons or antiprotons, which are confined states of first-generation (*u*, *d*) (anti)quarks. Quark masses attributed to the Higgs mechanism constitute only a few per cent of these hadronic masses, whereas the rest arises from dynamics of the strong interaction^28^. By contrast, in Mu atoms, the elementary muon and electron masses (*m*_μ_ ≈ 105.7 MeV *c*^−2^, *m*_e_ ≈ 0.511 MeV *c*^−2^) dominate over the (electromagnetic) binding energy (*Q*_em_ ≈ −13.5 eV). Hence, a Mu free-fall experiment would be a direct test of gravity acting predominantly on the mass content of the stress-energy tensor, and the only known feasible direct test using second-generation particles. Although mainly antimatter (antihydrogen) gravity was discussed on the theoretical side^23,29–31^, the free fall of Mu also offers to test flavour-dependent extensions to gravity^12^, as well as to probe beyond standard model interactions between electrons and muons^10^.

Carrying out this experiment is inherently challenging due to the short lifetime of μ^+^ (*τ*_μ_ ≈ 2.2 μs), and the difficulties to synthesize the atoms in vacuum. Measuring the small influence of gravity in such short timescales requires phase-sensitive methods like Talbot–Lau or Mach–Zehnder interferometry^32^, which is the proposed approach of the low-energy Mu interaction with gravity (LEMING) experiment^33^. Interferometry requires a Mu beam in vacuum, with high intensity and narrow angular and momentum distribution that conventional Mu sources could not provide. Before this work, vacuum Mu sources were based on porous materials such as SiO_2_ powder^34^ or aerogel^35^, in which muons could stop and recombine with electrons to form Mu. Typically, a few per cent of the formed Mu atoms reached the surface within their lifetime via diffusion through the nanoscopic pores^36^, which were emitted into vacuum with a thermal (Maxwell–Boltzmann) velocity distribution and broad ($\sim \cos \theta$) angular distribution^37^. Such thermal Mu beams would require drastic collimation and velocity selection for interferometry, which would reduce the number of atoms beyond feasibility for a gravity measurement. The low yield and large angular velocity spreads also introduce statistical and systematic uncertainties for precision Mu laser spectroscopy^38^. Although cooling these converters narrows the momentum distribution of the Mu atoms, the yields drop rapidly with temperature, with essentially no Mu diffusing to vacuum at below 100 K (ref. ^39^).

To overcome these limitations, the LEMING collaboration proposed a method to convert high-intensity μ^+^ beams to vacuum Mu using superfluid helium (He II)^33^, based on earlier muon moderation ideas^40^, and the microscopic behaviour of other exotic atoms in He II (ref. ^41^). Superfluid helium is known to be highly inert, repelling most impurities from its bulk. Mu as a light hydrogen isotope was also expected to have a large net positive chemical potential *E*_c_ when placed in He II (refs. ^40,42,43^). If other interactions can be neglected, Mu atoms that reach the liquid surface would be, hence, ejected with a uniform velocity $|{\mathbf{v}}| =\sqrt{2{E}_{{\rm{c}}}/{m}_{{\rm{Mu}}}}$ along the surface normal, broadened only by the diffusion velocity before emission. If He II is cooled below *T* < 0.3 K, the saturated vapour satisfies ultrahigh-vacuum conditions, and scattering of the ejected Mu atoms becomes negligible for interferometry and precision spectroscopy^33,34^.

This concept relied on several unknown factors, like the exact value of *E*_c_, the Mu formation probability below 0.7 K in He II (refs. ^44–46^), and most critically, the Mu thermalization and diffusion process. Mu atoms are created when muons come to rest in matter, in average ranges of *h* > 30 μm below the He II surface for conventional muon beams (${{\rm{p}}}_{{{\mu }}^{+}} > 12$ MeV *c*^−1^). For efficient vacuum Mu conversion, the average diffusion times *τ*_diff_ should not exceed the μ^+^ lifetime. Although this would not be possible from conventional bulk matter at *T* < 0.3 K, in He II, the mobilities are only limited by scattering on sparse thermal phonons and complex hydrodynamic effects like vortex nucleation for large impurities^47^. Theoretical predictions indicated that hydrogen and ^3^He isotopes^42^ and exotic atoms like antiprotonic helium^41^ cause small defects in He II. If Mu could be modelled as a similarly small impurity, a rapid (near-ballistic) diffusion could ensue without hydrodynamic drag, which was observed in the special case of ^3^He (ref. ^48^).

**Fig. 1:**
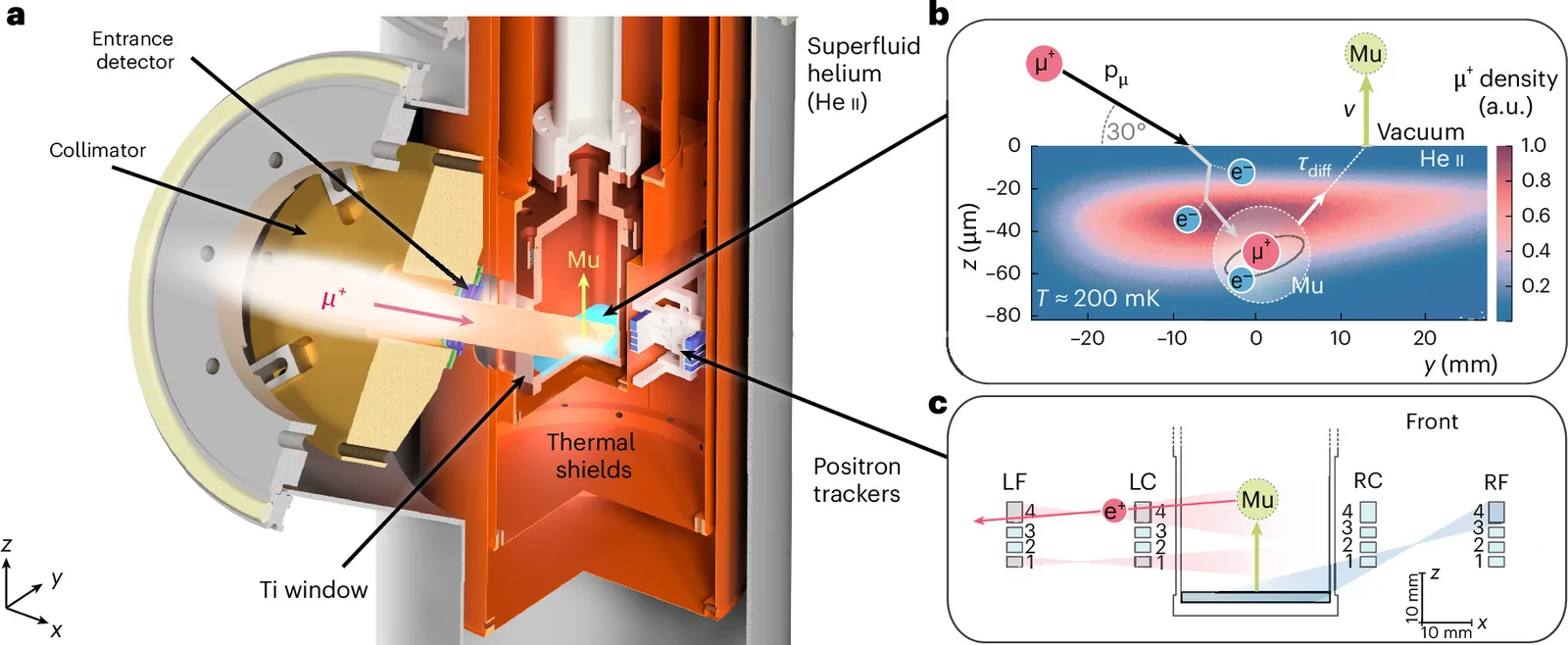
Experimental setup for observing Mu emission from He II. **a**, A collimated μ^+^ beam traverses an entrance detector and several thin foils before stopping in a thin horizontal layer of superfluid helium (He II). The He II target chamber is mounted to the mixing chamber plate of a dilution refrigerator and surrounded by three layers of thermal shields. **b**, Principle of Mu stopping and formation in He II at a temperature of *T* ≈ 200 mK. Incoming μ^+^ with momentum *p*_μ_ come to rest in an average 35-μm depth (coloured gradient in the background shows the simulated stopping distribution) and capture an electron from the ionization trail. The formed Mu diffuses ballistically to the surface over a time *τ*_diff_ and is emitted perpendicularly with a velocity *v* defined by the chemical potential. **c**, Schematic of the positron-tracking system used to localize Mu and μ^+^ decays within the target chamber. Detector segments (blue boxes) are labelled using their relative position to the He II target as left (L), right (R), close (C) and far (F) detectors. Shaded areas indicate the acceptance regions of selected detector coincidences: red areas denote coincidences of Layers 1 and 4 on the left side (LC1 ∧ LF1, LC4 ∧ LF4), whereas blue denotes a coincidence (RC1 ∧ RF4) monitoring the target liquid.

**Extended Data Fig. 1:**
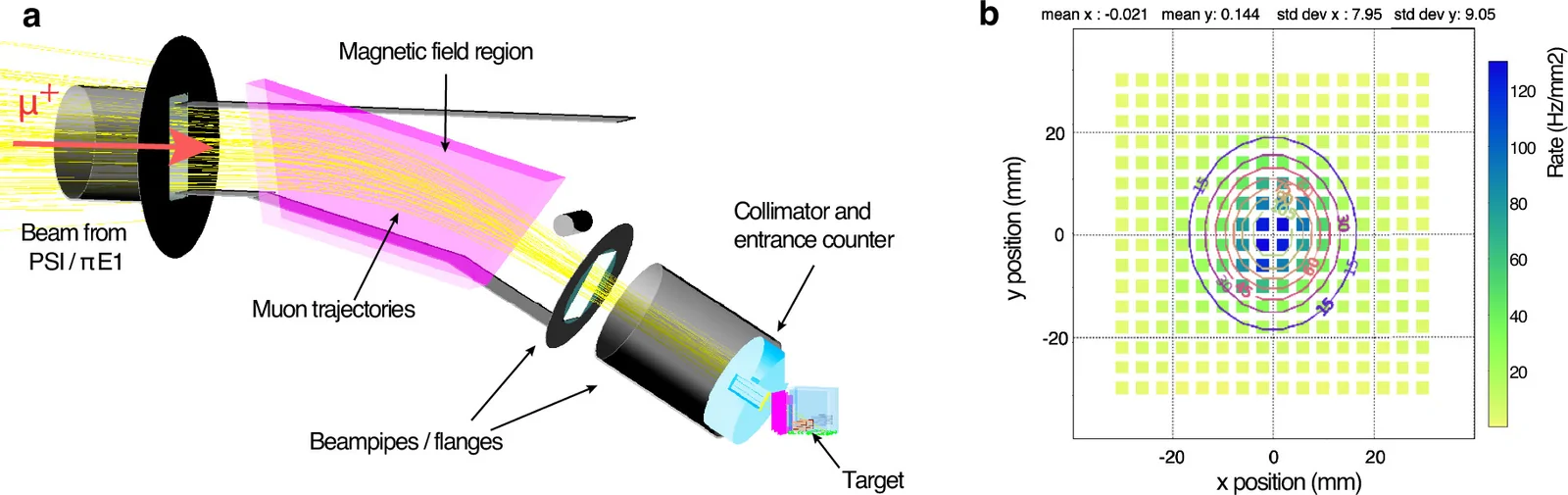
Simulation of the vertically bent beamline and the measured beam profile. (**a**) Schematic setup of the bent beamline used in the Monte Carlo simulations. Muon trajectories (yellow) from the position-momentum phase space of the *π*E1 beamline from PSI were sampled and propagated to minimize loss in the beam pipes (grey) while maximizing transmission to the target (blue). The magnetic field of the vertical bending magnet (pink) was obtained from finite element simulations. (**b**) Measured beam profile at the position of the entrance counter, using a retractable beam scanner with squared scintillator tiles read out by silicon photomultipliers. The circular lines and the annotations show contour lines of a 2D Gaussian fit to the rate in Hz/mm2. The beam waist was measured to be *σ* = 7.95 mm and 9.05 mm in the horizontal and vertical directions, respectively.

**Extended Data Fig. 2:**
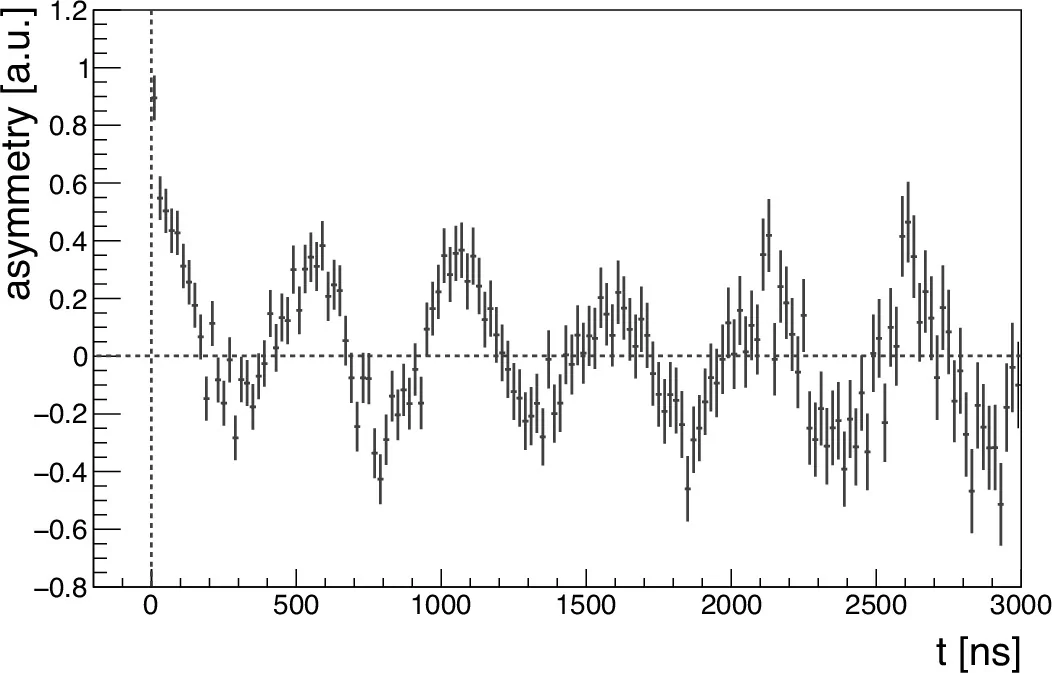
Muonium triplet precession in the target. Left-right asymmetry of C1 ∧ F4 coincidences, monitoring spin-dependent decays inside superfluid helium. The Larmor precession signal indicates that *μ*^+^ stopped in He II formed Mu atoms, and the early onset of the amplitude implies formation on a 100 ns timescale. Error bars denote ± 1*σ* statistical uncertainty.

To demonstrate Mu emission into vacuum from He II, the experimental setup (Fig. 1) was constructed at the Paul Scherrer Institute (PSI). A μ^+^ beam with momentum of *p*_μ_ = 12.5 MeV *c*^−1^ was bent downwards by a 30° angle (Extended Data Fig. 1). The beam traversed a 22-μm-thick scintillator foil that tagged the μ^+^ before entering the cryogenic chamber via a thin (6-μm) titanium window. The chamber was thermalized to *T* ≈ 0.2 K by a dilution refrigerator and filled with a 2-mm layer of isotopically purified He II. The μ^+^ beam momentum was tuned to optimize stopping close to the He II surface, in an average stopping depth of *h* = 35 μm (Fig. 1b and Methods). Although μ^+^ at rest cannot capture an electron from the ground-state ^4^He atoms at these temperatures, it could recombine with the last free electron from its ionization trail. We verified efficient Mu formation in the temperature range of 0.7–0.2 K with the observation of the Mu triplet spin precession (Extended Data Fig. 2), extending the range of earlier measurements^44^.

Mu emission from the liquid surface and propagation in vacuum was inferred by tracking positrons (e^+^) from muon decays (${\upmu }^{+}\to {e}^{+}{\nu }_{\mathrm{e}}{\bar{\nu }}_{\mu }$). For this purpose, a vertically segmented array of 16 plastic scintillator bars with silicon photomultiplier read-out^49^ was mounted on the two sides of the chamber (Fig. 1a,c). Coincidence conditions between detector segments defined geometric acceptance regions for the origin of decay positrons (Fig. 1c). Horizontal coincidences such as LC1 ∧ LF1 (red-shaded regions) were used to observe the time of Mu decays in increasing distances from the He II surface, whereas others like RC1 ∧ RF4 (blue-shaded area) monitored decays at the surface.

**Fig. 2:**
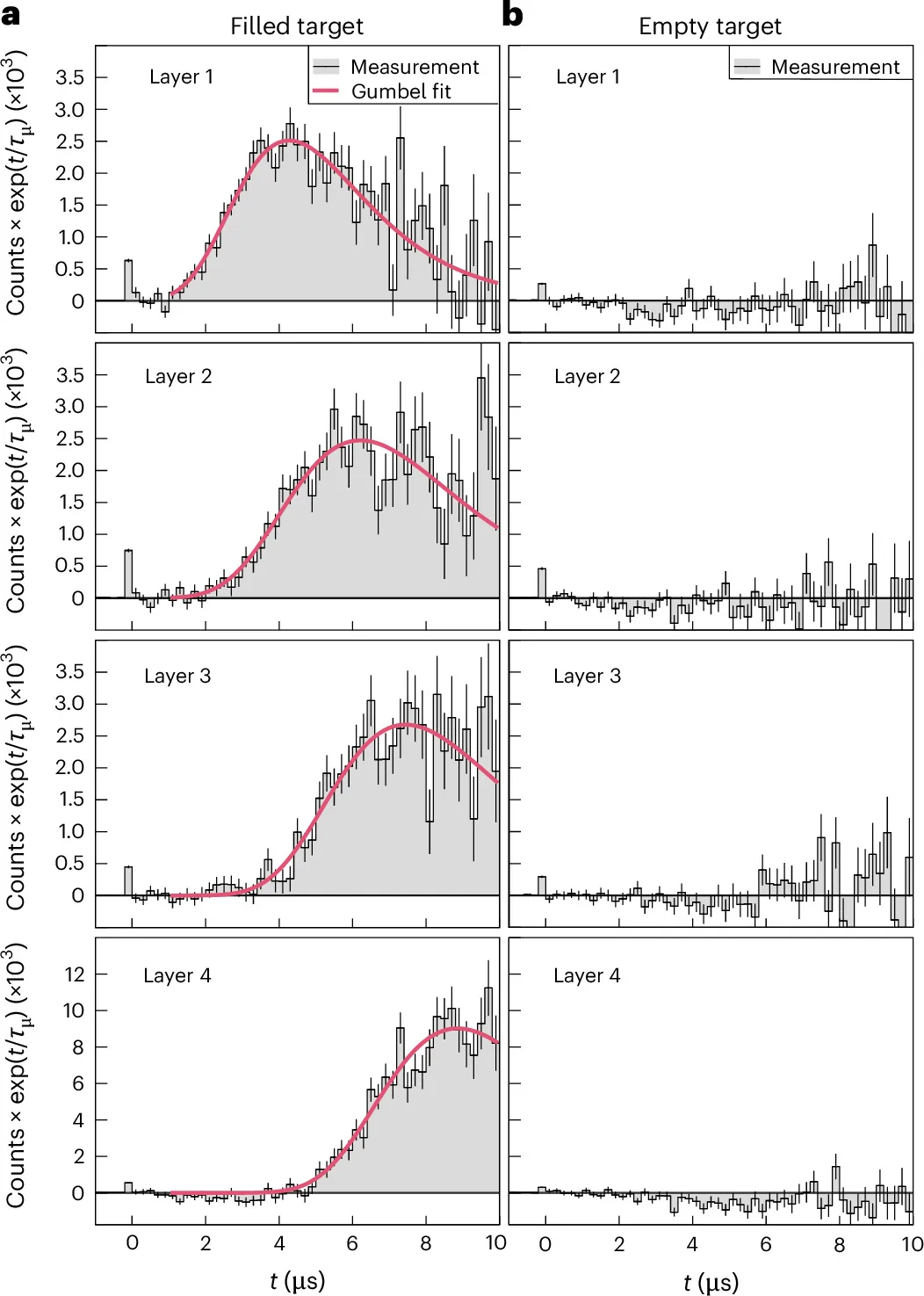
Time distribution across the four horizontal detector layers. **a**,**b**, Lifetime-compensated, background-subtracted time distributions for the He II target (**a**) and the empty target (**b**). For each horizontal layer (Fig. 1c), the corresponding left and right coincidence counts are summed (for example, LC1 ∧ LF1 and RC1 ∧ RF1) and scaled by ${e}^{t/{{\tau }_{\mu}}}$ to compensate the muon lifetime *τ*_μ_. **a**, A peak shifting systematically towards later times is observed in consecutive, more distant detector layers, implying Mu propagation. Centres of the peaks are extracted by a Gumbel test function fit to provide a model-independent estimate of the Mu propagation velocity. **b**, Flat distributions confirm the absence of Mu emission. Error bars represent ±1*σ* statistical uncertainties, propagated from the raw counts through lifetime scaling.

**Extended Data Fig. 3:**
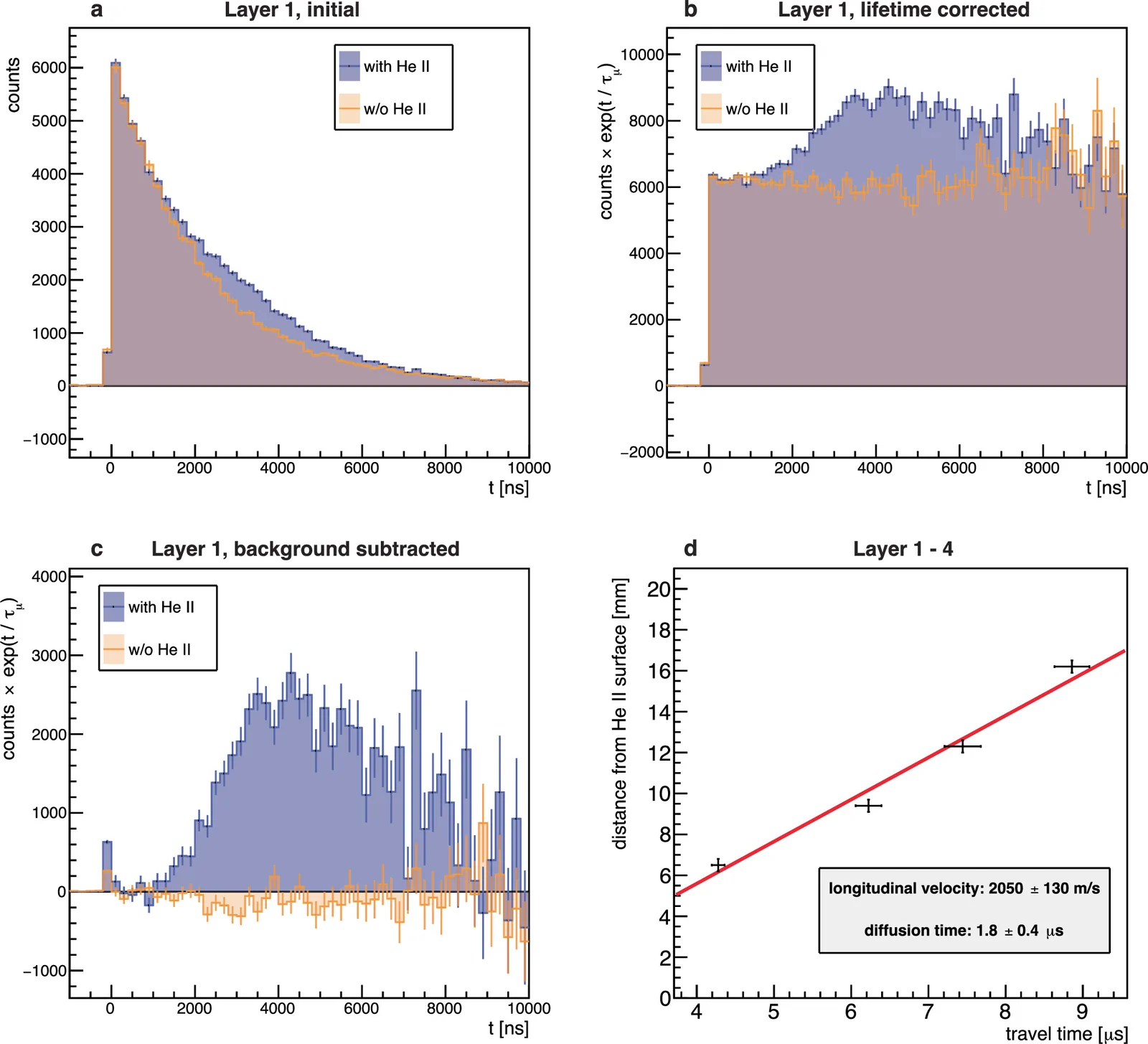
Model-independent time distribution analysis. The y-axis in panels (**a**-**c**) show the number of coincidence counts within a 200 ns interval as a function of time detected by Layer 1 for the He II target (blue) and empty background chamber (orange). Error bars denote ± 1*σ* statistical uncertainty. (**a**) Raw time distribution *N*_raw_ (t), rebinned from the digitizers, dominated by the characteristic exponential decay of *μ*^+^ with lifetime *τ*_*μ*_. (**b**) Lifetime-compensated distribution ${N}_{{\rm{lt}}}(t)={N}_{{\rm{raw}}}(t)\times \exp (t/{\tau }_{\mu })$, revealing a flat background with a peak arising from Mu dynamics. (**c**) Background-subtracted distribution *N*_final_ (t), where deviations from zero indicate Mu emission into vacuum. (**d**) Linear fit of the detector distance from the He II surface against the extracted mean arrival time, used to determine the propagation velocity and diffusion time of Mu. Horizontal error bars represent fitting uncertainty (Fig. 2a), while vertical error bars reflect detector positioning tolerances.

The resulting time distributions of μ^+^ decays in the four horizontal layers are shown in Fig. 2, where the time *t* = 0 was determined by the μ^+^ arrival on the entrance detector. In these histograms, the left and right coincidences were summed, and the counts and errors in each time bin centred around *t* were multiplied by $\exp (t/{\tau }_{\mu })$ to compensate for the effect of μ^+^ decays. Decays of μ^+^ in the target walls and floors appeared as a constant background, which was subtracted (Methods). Any deviations in the resulting time distributions indicate a dynamic behaviour, namely, Mu propagating through the acceptance regions. With He II present in the target (Fig. 2a), the time distribution of Layer 1 showed a clear rise between *t* = 2 μs and 4 μs, followed by a decline—indicating that Mu entered and exited the Layer 1 acceptance region above the liquid. In Layers 2–4, similar peak structures appeared at later times, indicating that Mu propagated upwards through the detection zones. By contrast, when the chamber was empty and μ^+^ stopped in the copper floor (Fig. 2b), a consistently flat distribution indicated no Mu emission. The time distributions (Fig. 2a) were first fit with a generic skewed Gumbel distribution to obtain a model-independent determination of the main characteristics of the emission. A linear fit of the peak centres versus the height of each layer above the He II surface yielded a preliminary estimate of the Mu propagation velocity of *v* ≈ 2.1 km s^−1^ in vacuum and a diffusion time of *τ*_diff_ ≈ 2 μs in He II (Extended Data Fig. 3).

For a detailed analysis, the measured time distributions were compared with Monte Carlo simulations (Methods). The above preliminary estimate on *τ*_diff_ is compatible with a fast, near-ballistic propagation model, without complex hydrodynamic effects that hinder the mobility of large impurities^47^. The effective diffusion velocity *v*_diff_ = *h*/*τ*_diff_ is hard to predict at *T* < 0.3 K due to the unknown thermalization and diffusion process. As discussed in the Methods, we simply assumed the existence of a limit velocity below which the ballistic propagation would take place. The Mu velocities in the liquid were, hence, sampled from flat distribution [0, 2 × *v*_diff_], with an isotropic direction from the formation point, defined by the stopping μ^+^. The average effective velocity *v*_diff_ was varied in the simulation. The effect of chemical potential at the surface was simulated by adding a uniform velocity ${\mathbf{v}}$ to the diffusion velocity in the direction of the surface normal, where the norm $| {\mathbf{v}} | =v$ was also varied in the simulation. A separate model assuming a thermal source with a Maxwell–Boltzmann velocity distribution and an ~cos*θ* angular distribution was also considered, with the temperature *T* varied in the simulation to find the best fit.

**Fig. 3:**
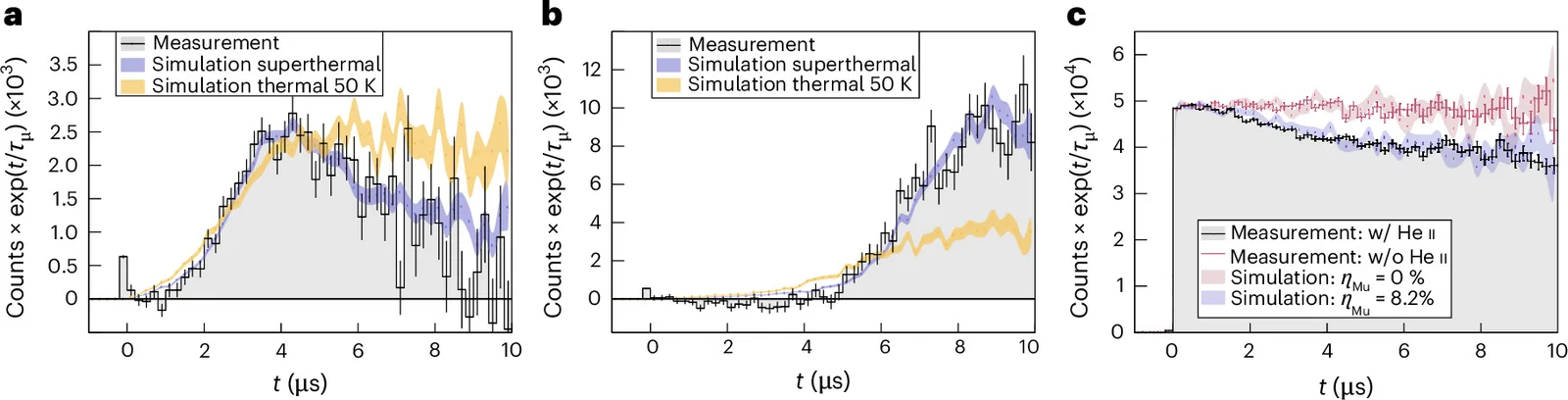
Comparison of measured and simulated time distributions. Examples of three measured time distributions (solid histograms) superimposed with the simulated ones (coloured bands) with ±1*σ* statistical uncertainties. **a**,**b**, Muon lifetime (*τ*_μ_) and background-compensated histograms show the Mu decays sampled with C1 ∧ F1 (Layer 1) coincidences close to the surface (**a**) and far from it (**b**) with C4 ∧ F4 (Layer 4) coincidence conditions. The simulated superthermal beam (blue) fits both distributions well, whereas even the best-fitting thermal beam (yellow) visibly deviates. **c**, Lifetime-compensated decays originating from the target bottom (C1 ∧ F4 coincidence conditions) when filled with He II (black) and when empty (red), superimposed with the corresponding simulations showing the escape of Mu from He II with an efficiency *η*_Mu_.

**Fig. 4:**
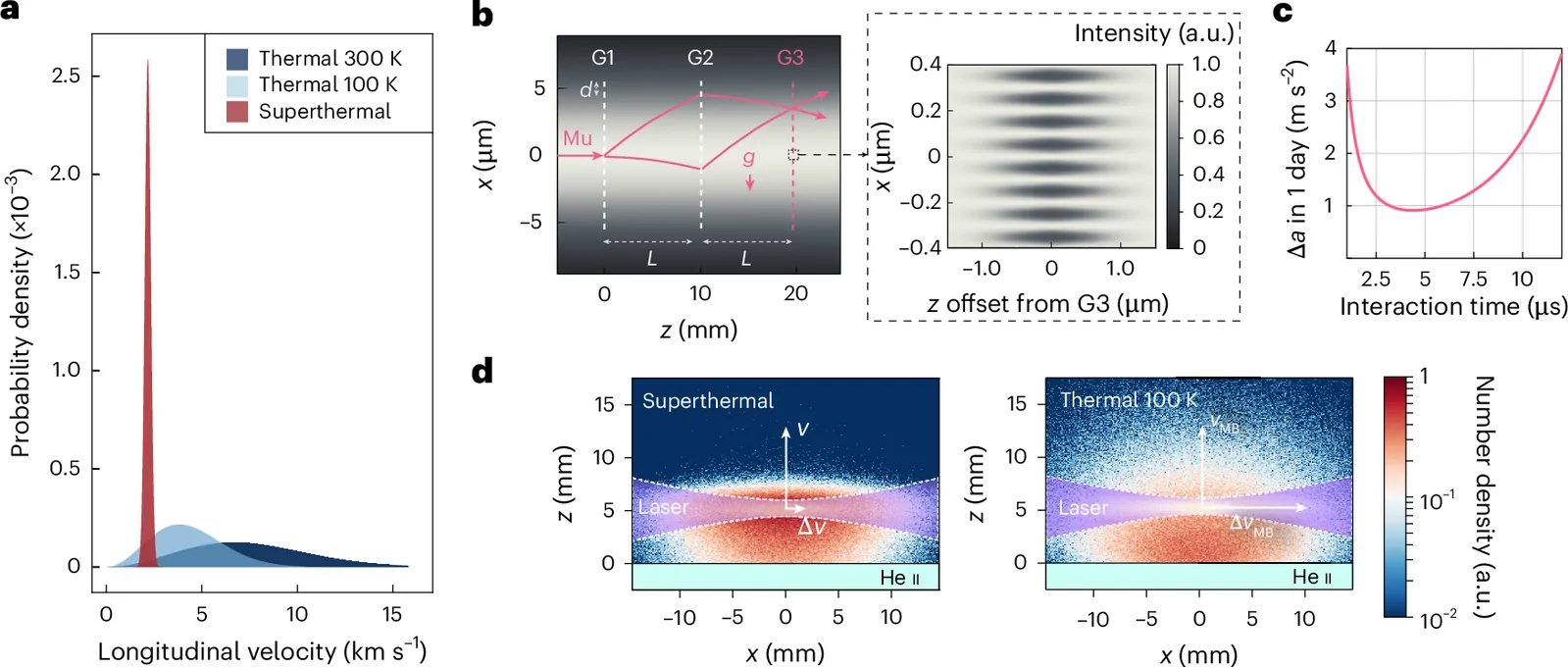
Beam characteristics of the superthermal beam and possible applications. **a**, Simulated velocity distributions of the superthermal Mu beam (red), based on the present experimental data, using the most pessimistic estimate on the velocity distribution (Δ*v* = 150 m s^−1^). As a reference, thermal (Maxwell–Boltzmann) distributions corresponding to conventional sources at room temperature (dark blue) and the coldest demonstrated (low-intensity) source at 100 K (light blue) are shown. **b**, Calculated intensity profile of the superthermal Mu beam traversing the interferometer (greyscale gradient). Diffraction on two gratings (G1 and G2) with pitch *d* and separation *L* create an interference pattern at the location of the third grating G3, which is vertically shifted due to the gravitational acceleration *g*. A zoomed-in view into G3 (right) shows the calculated intensity pattern without shift. **c**, Expected sensitivity to gravitational acceleration (Δ*a*) in 1 day at the πE5 beamline of PSI, assuming a grating pitch of *d* = 100 nm, as a function of interaction time *t*_*i*_. **d**, Simulated Mu distributions in vacuum (*z* > 0), 3.5 μs after the Mu formation, overlapping with a typical laser beam waist size. The superthermal beam (left) and a thermal source at 100 K (right) are shown assuming the same diffusion time. The arrows denote the longitudinal velocity and its standard deviation: (*v*, Δ*v*) for the superthermal and (*v*_MB_, Δ*v*_MB_) for the Maxwell–Boltzmann distributions.

**Extended Data Fig. 4:**
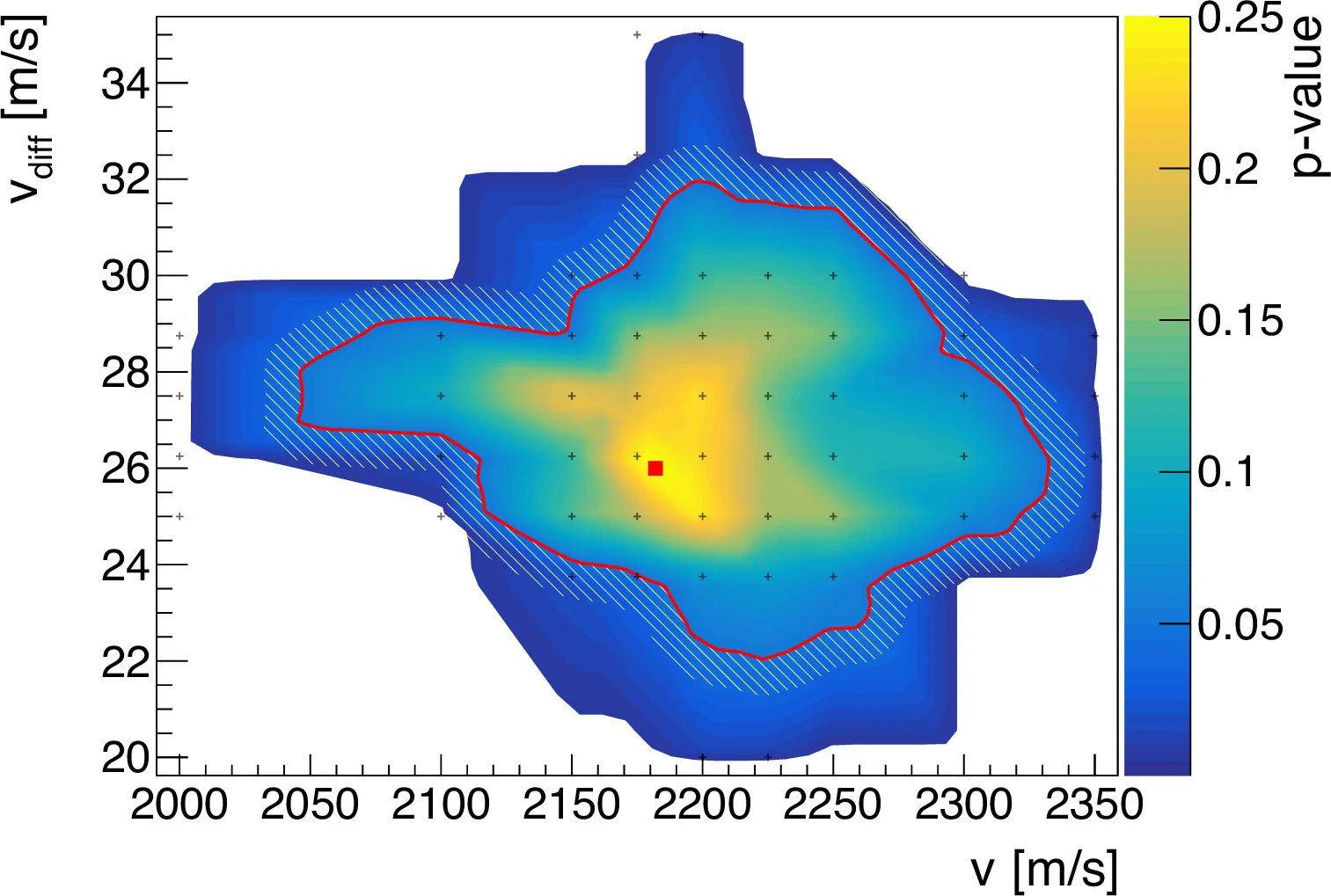
Parameter scan for the directed beam scenario. Mean diffusion velocity *v*_diff_ and longitudinal vacuum propagation velocity v, are varied (simulated values are indicated by black crosses), and the p-value from the *χ*^2^ test of homogeneity between measured and simulated time distributions are plotted. The red square marks the best fit and the red line shows the p-value = 0.05 boundary corresponding to a two-sided 90% confidence interval. The dashed region marks the excluded side.

Figure 3 shows the measured time distributions (black histogram) alongside the two simulated beam models for Layers 1 (Fig. 3a) and 4 (Fig. 3b), with the best-fitting parameters. A combined *χ*^2^ across all four horizontal layers quantified the goodness of simulation under the given parameters^50^. The best-fitting thermal beam model (yellow band) was found at *T* = 50 K, but it still failed to give a good reproduction of the data (reduced *χ*^2 ^= 1.98, with the number of degrees of freedom (ndf) = 459), whereas the best-fitting superthermal beam model (blue) closely matched it (reduced *χ*^2^ = 1.03, ndf = 459). These results were obtained by a parameter sweep that yielded a longitudinal propagation velocity of $v=(218{0}_{-130}^{+160})$ m s^−1^ and an effective diffusion velocity of ${v}_{{\rm{diff}}}=(2{6}_{-4}^{+6})$ m s^−1^, with statistical uncertainties corresponding to a 90% confidence region (Extended Data Fig. 4). Assuming the longitudinal velocity originates purely from the chemical potential we obtained *E*_c_ ≈ 2.8 meV. The above results were validated using an independent dataset with a lower liquid level, yielding consistent results. The uncertainties were driven mainly by statistics and the detector resolution, and were largely insensitive to changes in transverse velocities due to the one-dimensional tracking geometry. We assessed the sensitivity of the simulated time distributions to experimental uncertainties, such as initial μ^+^ beam parameters, degrader thicknesses and detector positions. In case of the mean diffusion velocity *v*_diff_, this contributed to an additional *δv*_diff_ ≈ 15 m s^−1^ systematic uncertainty, whereas the uncertainties of the propagation velocity *v* remained unaffected.

**Extended Data Fig. 5:**
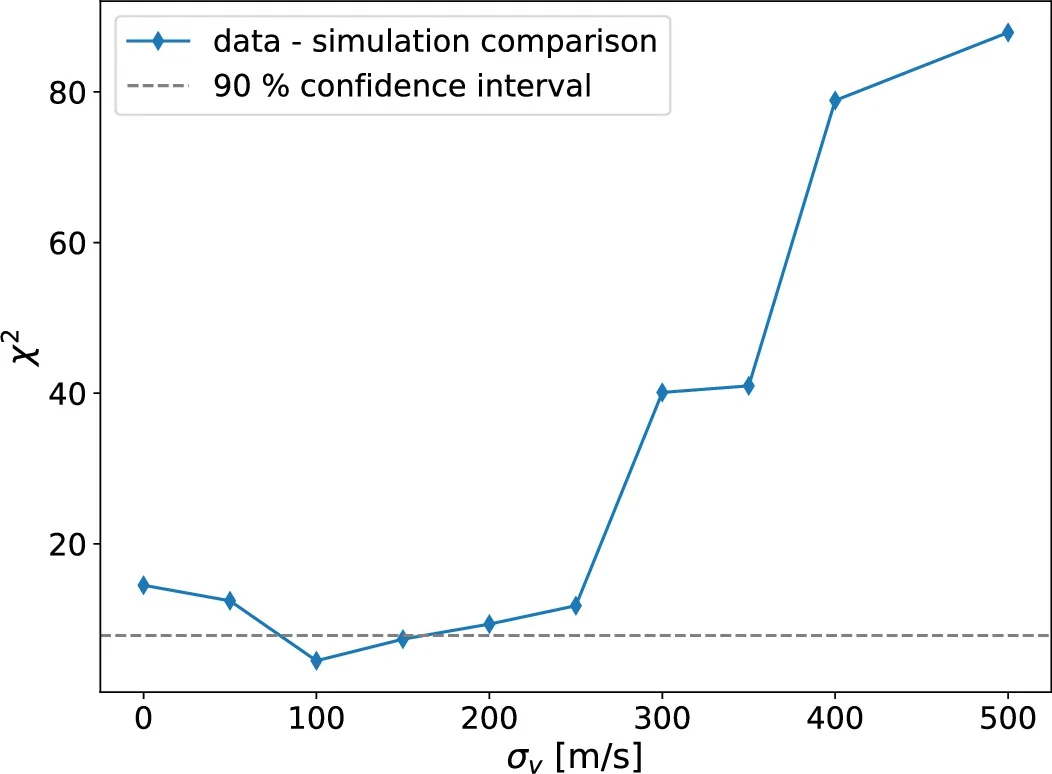
Estimation of velocity distribution. *χ*^2^ obtained from comparing the shape of the time distributions of simulated Mu beams with defined velocity broadening *σ*_*v*_ and the measurement. Velocity spreads of 80-150 m/s are within a 90% confidence interval. The solid line is drawn as a guide to the eye.

The diffusion velocity in He II provided a lower bound for the velocity spread (Δ*v* ≥ *v*_diff_) and the angular divergence (*α* ≥ *v*_diff_/*v* ≈ 12 mrad) of the emitted beam. Interactions at the surface (like scattering on impurities or surface waves) may result in additional broadening of the vacuum velocity distribution, which we investigated in simulations by comparing the rising edges of the time distributions. An isotropic velocity spread in the range of (80 < Δ*v* < 150) m s^−1^ was found to be consistent with the data (Extended Data Fig. 5). Even using the conservative upper limit for Δ*v* (~150 m s^−1^) (Fig. 4a), the beam is clearly superthermal (Δ*v* ≪ *v*).

The fraction of emitted Mu atoms with respect to the stopped muons in He II ${\eta }_{{\rm{Mu}}}={{\rm{Mu}}}_{\mathrm{vac}}/{\upmu }_{{\rm{stop}}}^{+}$ was determined from monitoring decays in the target using the C1 ∧ F4 coincidences (Fig. 3c). The decrease in counts in the measured curve indicated Mu atoms leaving He II, and decaying outside the geometric acceptance of the detectors. The amount of Mu leaving the conversion target was counted, and also modelled in the Monte Carlo simulation (Fig. 3c, coloured bands), which yielded a conversion efficiency of ${\eta }_{{\rm{Mu}}}=(8.{2}_{-1.1}^{+0.8})\, \%$ (reduced *χ*^2^ = 1.13, ndf = 99) (Methods).

Using the muon rates *R*_μ_(*p*_μ_) of the highest-intensity (πE5) beamline of PSI at the same *p*_μ_ = 12.5 MeV *c*^−1^ momentum as the present study, the projected Mu rates can be estimated using the above conversion efficiency *R*_Mu_ = *η*_Mu_ × *R*_μ_. The superthermal source data compared with the projected performance of existing vacuum Mu sources are summarized in Table 1 (Methods). The He II target yields vacuum Mu rates comparable with the best-performing room-temperature aerogel targets, whereas its longitudinal velocity distribution (Fig. 4a) is an order of magnitude narrower than the coldest (low yield) thermal sources at 100 K.

**Table 1:** Comparison of vacuum Mu sources

Target	*p*_μ_ (MeV *c*^−1^)	*R*_μ_ (s^−1^)	*η*_Mu_ (%)	*R*_Mu_ (s^−1^)
Aerogel (300 K)^35^	23	1.1 × 10^7^	3.05 ± 0.03	(3.35 ± 0.03) × 10^5^
SiO_2_ film (100 K)^39^	1	3.0 × 10^3^	20 ± 4	(6.0 ± 1.2) × 10^2^
He II (0.2 K) (this work)	12.5	1.2 × 10^6^	8.2 ± 1.0	(9.8 ± 1.2) × 10^4^

Projected Mu rates *R*_Mu_ = *η*_Mu_ × *R*_μ_ from various converters used at the optimum μ^+^ momentum (*p*_μ_) at which the Mu emission efficiency per stopped muon, *η*_Mu_, was measured. The μ^+^ rates *R*_μ_(*p*_μ_) are based on the PSI πE5 (for aerogel and He II) and LEM (for SiO_2_ film) beamlines, collimated to a 10 × 10-mm^2^ aperture.

The feasibility of a Mu gravity experiment depends strongly on the parameters of the atomic beam. Considering a generic three-grating interferometer (Fig. 4b), with *L* distance between the gratings, diffraction on the first two gratings (G1 and G2) creates interference fringes at the position of the third (G3). Gravitational acceleration shifts this pattern vertically $\Delta x={g}_{{\rm{Mu}}}{t}_{i}^{2}/2$, where *t*_i_ = *L*/*v* is the time of flight between the gratings. This phase shift can be measured by counting the transmitted Mu atoms as a function of the vertical displacement of G3 (ref. ^33^). The smallest observable acceleration (Δ*a*) can be expressed as^32^

1 $$\Delta a=\frac{1}{2\uppi {t}_{{\rm{i}}}^{2}}\frac{d}{C\sqrt{N}}$$

where *C* is the contrast of the interference pattern, *d* ≈ 100 nm is the grating period chosen from fabrication constraints^51^ and *N* stands for the total number of detected atoms after G3. Starting from *N*_0_ atoms, μ^+^ decays limit the detected number as $N\propto {N}_{0}{e}^{-2{t}_{{\rm{i}}}/{\tau }_{\upmu }}$, which defines an optimum interaction time for the highest sensitivity *t*_*i*_ ≈ 4.5 μs (Fig. 4c), and the corresponding grating spacing *L* = 9.6 mm.

Conservative calculations based on partially coherent waves^52^ predict a high fringe pattern with a contrast *C* > 0.35 over an extended ~1-μm region along the optical axis, which is amenable for grating alignment (Methods). The small angular divergence also minimizes aperture-related losses, which would be prominent using thermal beams. Combined with the expected Mu rate of *R*_Mu_ ≈ 1 × 10^5^ s^−1^ at PSI, this source adapted to a vertical geometry would enable a measurement of the gravitational acceleration with Δ*a*/*g* ≈ 0.01 relative precision within ~100 days of measurement. Further improvements are expected by ongoing muon beam developments at PSI, which would enable up to two orders of magnitude increase in surface muon rates.

With regard to Mu 1S–2S laser spectroscopy, a high number density of Mu atoms within the laser beam waist diameter *ω* < 1 mm is critical. Simulated spatial distributions of Mu atoms 3.5 μs after the muon arrival (Fig. 4d) show the superthermal beam and a 100-K thermal source from porous SiO_2_ proposed for the latest spectroscopy experiments^5^, using the same diffusion time for fair comparison. While a thermal source is rapidly dispersing, the superthermal beam forms a compact, non-dispersive cloud only broadened by the diffusion time differences. Scaling with the rates shown in Table 1, nearly 10^3^ times more Mu atoms could be addressed by the laser using the superthermal source. The lower velocity of the atoms would also decrease transit-time broadening (∝*v*/*ω*) by a factor of two, whereas the narrow velocity distribution would reduce the main uncertainty in second-order Doppler shift corrections (∝*v*Δ*v*) by an order of magnitude. These advantages, combined with state-of-the-art excitation schemes^5^, could push the precision of the 1S–2S transition frequency measurement to the sub-kilohertz level, allowing a precise determination of the muon mass and tests of bound-state quantum electrodynamics. The present efforts of the LEMING collaboration are directed towards exploiting the high rates and increased beam quality to demonstrate Mu interferometry in a subsequent per-cent-level precision test of the weak equivalence principle using second-generation standard model particles. The applications of the superthermal beam in searches for Mu–anti-Mu oscillations or for the synthesis of low-energy muons are also investigated.

## Methods

### Design of beamline and cryogenic converter

Positive muons from the low-intensity πE1 beamline^53^ of the CHRISP (Swiss Research Infrastructure for Particle Physics) facility of PSI were delivered using a dedicated beamline segment with a 30° vertical downward bend. The magnetic elements and beam propagation were optimized using COMSOL Multiphysics (https://www.comsol.com) and Geant4 packages^54,55^ (Extended Data Fig 1a). The vertical bending magnet designed for this purpose had 12 × 6 turns of laminated coils carrying up to 60-A current and an H-type yoke that defined a trapezoid magnetic field. The aperture width of ~80 mm and an average field length of 40 cm were a compromise between the magnetic field quality and particle transmission. The collective focusing power of the last πE1 quadrupole and the trapezoid dipole magnetic field resulted in a nearly symmetric two-dimensional Gaussian beam profile. This profile was measured and optimized using a retractable beam scanner, resulting in a horizontal (vertical) beam waist of *σ* = 7.95 (9.05) mm, and a rate of *R*_μ_ ≈ 1.5 × 10^4^ μ^+^ s^−1^ at *p*_μ_ = 12.5 MeV *c*^−1^(Extended Data Fig. 1b).

The μ^+^ beam was passing through a 40-mm-thick brass collimator placed in front of the cryogenic chamber, with a 12 × 25-mm^2^ vertical aperture. The muon entrance detector that tagged the incoming μ^+^ was mounted to the exit of this collimator. It was composed of a (22 ± 7)-μm-thick plastic scintillator foil (EJ-212), with the edges glued to an acrylic frame that efficiently coupled out the weak scintillation light to eight silicon photomultipliers (AdvanSiD SiPM). The tagged muons traversed three layers of thermal shielding with ~2-μm-thick aluminized Mylar foils covering apertures, before they entered the target chamber via the (6.0 ± 1.5)-μm-thick titanium beam window. The pressure inside the target chamber was given by the extremely low saturated vapour pressure of ^4^He, which is expected to be *p* ≈ 10^−15 ^mbar at *T* = 0.2 K (ref. ^56^). With this, the scattering of emitted Mu atoms could be neglected^34^.

The central momentum *p*_μ_ and momentum bite Δ*p*_μ_/*p*_μ_ ≈ 1.5% (full-width at half-maximum) was set by scaling magnet currents and adjusting slits in the beamline following predefined values for surface muon beams, which was prone to uncertainties. The stopping power carried additional uncertainties from material thicknesses of the titanium foil and the entrance detector described above. Experimental ranging of the μ^+^ beam momentum was, hence, carried out by tuning the magnet currents and monitoring the number of μ^+^ decays in the target, where a sudden increase indicated the limit ${p}_{\upmu }^{0}$ at which the beam passed the degrader layers. The measured ${p}_{\upmu }^{0}$ was 0.3 MeV *c*^−1^ smaller compared with expectations from simulations, which is well within the above uncertainties. In further simulations, the corrected *p*_μ_ = 12.5 MeV *c*^−1^ experimental momentum and the nominal degrader thicknesses were used to obtain the stopping distribution (Fig. 1b), resulting in the mean depth of *h* = 35 μm. The ranging uncertainties yield an uncertainty of *δh* ≈ 15 μm, which was taken into account in estimating systematic uncertainties of the diffusion velocity.

The He II target was contained in an oxygen-free high-conductivity copper chamber mounted onto a custom-built dilution cryostat^57^ with tail and shielding optimized for this experiment. The side walls of the chamber were thinned down towards the detector array to <1 mm to minimize scattering of the decay positrons. The target gas was isotopically pure ^4^He with an isotopic ratio of ^3^He/^4^He ≈ 10^−12^, to enhance Mu diffusion and to avoid the accumulation of ^3^He impurities at the surface. This isotopic purification was vital, since our previous attempt using high-purity (99.9999%, 6N) commercially available helium gas with natural abundance (^3^He/^4^He ≈ 10^−6^) did not result in vacuum emission, which was accounted for Mu scattering on ^3^He.

The ^4^He gas was iteratively measured and filled from a room-temperature buffer volume of 1.0 l and delivered to the chamber by two capillaries. With this method, the depth of the liquid surface could be adjusted with ±0.3-mm precision. The temperature was measured with 5-mK precision using two calibrated Cernox sensors from Lake Shore Cryotronics, attached to the top and bottom of the chamber. Since the exact temperature below *T* < 0.3 K was less relevant for the observed phenomenon, the cryostat was allowed to run free at the base temperature, which drifted in time between *T*_top_ ≈ 170–195 mK and *T*_bot_ ≈ 225–235 mK on the two sensors.

### Observation of Mu formation at 200 mK

Mu formation was observed by their triplet precession in the weak residual (~50−100 μT) magnetic fields, dominated by the Earth’s magnetic field^58^. Positive muons emit decay positrons preferentially in the direction of their spin; hence, spin precession of the initially polarized fraction of muons or Mu atoms appear as an oscillatory signal in the surrounding detectors. This signal can be enhanced by looking at the time-dependent asymmetry (*N*_l_ − *N*_r_)/(*N*_l_ + *N*_r_) between left-sided (*N*_l_) and right-sided (*N*_r_) detector counts using LC1 ∧ LF4 and RC1 ∧ RF4 coincidences (Extended Data Fig. 2). At such low fields, the Mu triplet precession *ω*_Mu_ is only visible in microsecond timescales, being considerably faster than the muon precession, *ω*_μ_ ≈ *ω*_Mu_/105. The early appearance of asymmetry implied that most of the Mu atoms formed promptly on stopping, or on timescales of *τ*_μ__→Mu_ ≪ 1/*ω*_Mu_, which agrees with earlier observations made at higher temperatures^46^.

A quantitative muon-spin resonance measurement would have needed a well-controlled magnetic field, but relative changes in the amplitudes were monitored and no notable change was seen below 0.7 K. This indicated that formation efficiencies were not changing drastically at low temperatures from the previously measured ~90% at 0.7 K (ref. ^44^), which agreed with the lower limit of the formation efficiency (~60%) implied from the present emission measurements too (see below).

### Processing of time distributions

The time distributions shown in Fig. 2 were constructed from coincidence counts between a close and far detector within a 40-ns coincidence window, where *t* = 0 (muon arrival) was defined by the entrance counter. Distributions of the left *N*_l_(*t*) and right *N*_r_(*t*) detector pairs were summed to increase the solid angle, and to cancel the effects of spin precession: *N*_raw_(*t*) = *N*_l_(*t*) + *N*_r_(*t*) (Extended Data Fig. 3a). The counts and statistical error bars in each time bin centred around time *t* were multiplied by $\exp (t/{\tau }_{\mu })$ to compensate for the muon decay: ${N}_{{\rm{lt}}}(t)={N}_{{\rm{raw}}}(t)\times {e}^{t/{\tau }_{\mu }}$. In these lifetime-compensated distributions, the decays from μ^+^ in the target walls and floors appear as a constant background starting at *t* = 0 (Extended Data Fig. 3b). For Layers 1–4, the constant muon background was calculated by averaging the counts in the first 1 μs, where no Mu atoms were expected within the detector acceptances. The background, ${N}_{\mathrm{bg}}=\frac{1}{N}{\sum }_{t=0}^{t=1\,\upmu {\rm{s}}}{N}_{\mathrm{lt}}(t)$, with *N* denoting the number of bins in the 0–1-μs window, was subtracted from the lifetime-compensated distributions to obtain the distributions containing only the vacuum Mu signal *N*_final_(*t*) = *N*_lt_(*t*) − *N*_bg_ (Extended Data Fig. 3c). The histograms were rebinned only after this last step to a 200-ns width.

To determine the main characteristics of emission in a model-independent way, the resulting distributions were fit with a skewed test function (a Gumbel distribution) that had the least degree of freedom and described the data well (reduced *χ*^2^ = 1.24, 1.26, 1.02 and 1.57 for Layers 1–4, respectively; ndf = 42). The fit function was $f(t)={f}_{0}{e}^{-(z+{e}^{-z})}$, with $z=\frac{t-\gamma }{\beta }$, and the fit parameters *f*_0_, *γ* and *β* denote the peak amplitude, peak position and width, respectively. The peak position *γ* provided the mean travel time of Mu to each layer and is plotted against the height above the liquid surface (Extended Data Fig. 3d). The slope of a linear fit yielded a preliminary estimate of the Mu propagation velocity in vacuum (~2.1 km s^−1^), whereas the time offset at zero distance from the liquid surface yielded a diffusion time in He II (~2 μs). These values were used for the initial parameterization of the Monte Carlo simulations, where we carried out a detailed analysis by varying them.

For the Mu emission plot using C1 ∧ F4 coincidences that monitor the liquid surface (Fig. 3c), the background was not subtracted, since the signal investigated here is the escape (as opposed to the appearance) of Mu from the target.

The number of escaping Mu atoms against all stopped muons (the conversion efficiency *η*_Mu_) was also determined in a model-independent way from the original time distribution by counting the missing decays in late time, under the exponential decay curve fitted to the first time bins (*t* < 1 μs). This method yielded *η*_Mu_ = (8.6 ± 0.8)%, consistent with optimized results from the Monte Carlo simulations.

### Simulation of Mu diffusion and emission

The Mu beam emitted from He II was modelled using the Geant4 framework^54,55^. First, the stopping distribution of μ^+^ in He II was simulated using experimental ranging data, as described above, and taken as the starting position of the Mu atoms.

With the lack of a microscopic model, the thermalization and diffusion in He II was modelled based on simple considerations and the measured transport properties of other impurities. The initially hot Mu atoms were assumed to thermalize by creating collective excitations, until they reach the kinematic limit defined by the dispersion relation, featuring the roton minimum (Δ*E*_rot_/*k*_B_ ≈ 8.6 K, *p*_0_ ≈ 1.9 Å^−1^). For a heavy particle, this limit would be the Landau velocity (*v*_L_ ≈ 60 m s^−1^), but the exact dynamics is sensitive to the effective mass of the impurity and complex hydrodynamic effects. Most atoms and negative ions cause large (nanometre-sized) defects in He II, and hydrodynamic backflow gives rise to large effective masses (${m}^{*} > 50\,{m}_{{4\atop}{\rm{He}}}$, with ${m}_{{4\atop}{\rm{He}}}$ being the atomic mass of ^4^He)^47,59^. For ions, the ensuing hydrodynamic effects like vortex nucleation limit the effective velocities in He II to ${v}_{\max } < 1$ m s^−1^ (ref. ^47^) under saturated vapour pressure, which would not allow Mu atoms to escape He II within their lifetime. Defects caused by Mu atoms were, on the other hand, expected to be much smaller, ${m}_{{\rm{Mu}}}^{* }\approx 2.5\,{m}_{{4\atop}{\rm{He}}}$, based on the calculated and measured properties of hydrogen and helium isotopes^42,60^. This value is closer to the effective mass of ^3^He in He II $({m}_{{3\atop}{\rm{He}}}^{* }\approx 1.5{m}_{{4\atop}{\rm{He}}})$, which is known to exhibit fast (ballistic) diffusion, scattering only on thermally available phonons^48^. The scarcity of phonons at *T* < 200 mK (*ρ*_ph_ ∝ *T*^3^) (ref. ^48^) and ^3^He impurities in the purified liquid make collisions during the observed microsecond-scaled diffusion times unlikely.

On the basis of these considerations, we simulated the diffusion of Mu in He II as ballistic propagation with an upper velocity limit. The magnitude of the velocity of each Mu atom was sampled from a flat distribution [0, 2 × *v*_diff_], with the direction isotropically distributed from the point of origin defined by the μ^+^ stopping position. The average diffusion velocity of the ensemble *v*_diff_ was a variable parameter in the simulation.

Mu emission into vacuum was modelled by adding an additional velocity vector ${\mathbf{v}}$ to the above-determined diffusion velocity ${\mathbf{v}}_{{\rm{diff}}}$. Multiple scenarios were considered, with two presented in this work. (1) Superthermal beam: a constant ${\mathbf{v}}$ was added in the direction of the surface normal, motivated by the assumption that Mu gains a (mono-energetic) kinetic energy from the positive chemical potential on leaving the flat surface of He II. (2) Thermal beam: we chose the Maxwell–Boltzmann velocity distribution that fit the overall experimental data the best (*T* ≈ 50 K), and directed it with a random sample from a $\cos \theta$ angular distribution, where *θ* is the emission angle with respect to the surface normal. The resulting Mu trajectories were propagated above the surface, and the detector response from subsequent decay positron trajectories were used to generate the simulated time distributions, which we compared with the measured ones.

The directed beam model included two free parameters: the average diffusion velocity *v*_diff_ and the longitudinal velocity *v* acquired on surface ejection. We conducted a two-dimensional parameter scan, where for each pair, the corresponding *p* value from a *χ*^2^ test comparing simulated and measured time distributions was calculated^50^. The best-fit values correspond to the maximum *p* value and we determined the uncertainties as the region where *p* = 0.05, representing a 90% confidence region. This yielded an average diffusion velocity of ${v}_{{\rm{diff}}}=(2{6}_{-4}^{+6})$ m s^−1^ and longitudinal velocity of $v=(2,180_{-130}^{+160})$ m s^−1^ (Extended Data Fig. 4).

To obtain the vacuum Mu yield from the simulation as well, we implemented a formation efficiency 0 ≤ *ϵ*_Mu_ ≤ 1 as a free parameter, which limits the fraction of Mu atoms that undergo the diffusion process and do not decay at the formation position. With *v*_diff_ = 26 m s^−1^ and *v* = 2180 m s^−1^, a minimal *χ*^2^ between the measured and simulated *C*1 ∧ *F*4 time distribution was obtained for ${\epsilon }_{{\rm{Mu}}}=6{0}_{-8}^{+6} \%$. Taking into account the lifetime losses during the diffusion process and that only half of the atoms diffuse towards the surface, this yields a vacuum Mu efficiency of $(8.{2}_{-1.1}^{+0.8})\, \%$, in agreement with the model-independent estimations.

### Estimation of velocity distribution beyond thermal contributions

To assess the influence of possible surface interactions on the emergent Mu velocity distribution, independent simulations of Mu emission were performed, assuming, for a generic description, a Gaussian velocity profile centred at 2,180 m s^−1^ with variable isotropic velocity spreads of *σ*_v_. A large velocity distribution would cause the expansion of the cloud of atoms, and would appear as a change in the rising edge in consecutive detector layers. For each *σ*_v_, simulated time distributions were compared with the measured ones by calculating a *χ*^2^ from the steepness of the rising edges in Layers 2–4 relative to Layer 1. Velocity spreads in the range of *σ*_v_ ≈ 80–150 m s^−1^ were found to be consistent with the data at the 90% confidence level (Extended Data Fig. 5), with *σ*_v_ ≈ 100 m s^−1^ being the best fit. For the evaluation of beam quality, we chose the conservative upper limit of *σ*_v_ < 150 m s^−1^, and assumed isotropic velocity spreads in all directions.

### Sensitivity calculations for interferometry

The preliminary estimate of sensitivity is based on simple considerations of a phase measurement in a three-grating interferometer (for example, see ref. ^32^). Besides decay losses, the number of detectable atoms *N* is further reduced by geometric factors, $N={N}_{0}\alpha {\vartheta }_{\mathrm{G}}^{3}{\eta }_{\mathrm{D}}\times {{\rm{e}}}^{-2{t}_{i}/{\tau }_{\upmu }}$. Here the acceptance (*α*) of the interferometer accounts for the fraction of atoms that could pass the 1-cm^2^ aperture of the first and third gratings. Using a simple Monte Carlo model, thermal (Maxwell–Boltzmann) beams with $\sim \cos \theta$ angular distribution suffer large losses here, with *α*_th_ ≈ 0.07 for the 4.5-μs optimum time-of-flight length (${\overline{v}}_{\mathrm{th}}\approx 6,600$ m s^−1^, *L*_th_ ≈ 29 mm). By contrast, the superthermal beam is expected to have *α* ≈ 0.95 acceptance. The transmission of a single grating (*ϑ*_G_ ≈ 0.35) and detection efficiency (*η*_D_ ≈ 0.75) further decrease the measured *N*, where the above estimates are based on the geometry of the gratings and estimated solid angle coverage of the detectors.

The expected contrast ($C=\frac{{I}_{\max }-{I}_{\min }}{{I}_{\max }+{I}_{\min }}$), defined by the intensity minimum (${I}_{\min }$) and intensity maximum (${I}_{\max }$) of the interference pattern, was calculated using mutual intensity functions from partially coherent wave optics, and a Gaussian Shell model of the particle beam^52^. The atoms have a de Broglie wavelength of *λ*_Mu_ ≈ 1.5 nm, which results in a Talbot length of ${L}_{{\rm{T}}}=\frac{2{d}^{2}}{{\lambda }_{{\rm{Mu}}}}\approx 14\,\upmu {\rm{m}}$. This would put the optimum grating spacing in the far-field regime (*L* ≫ *L*_T_). On the other hand, the large aperture size does not allow for the separation of the main diffraction orders at *L* distance, meaning the second grating is still placed in the aperture near field.

A typical beam waist size of a focused muon beam defined the initial beam width as *A*_0_ = 10 mm. The transverse coherence width *ℓ*_0_ of the superthermal source was estimated conservatively from the upper limits of the angular divergence *α* ≈ Δ*v*/*v* ≈ 70 mrad, using the van Cittert–Zernike theorem *ℓ*_0_ ≈ *λ*/*α* = 22 nm. The wavefront curvature *r*_0_ of the superthermal source was assumed to be large compared with the length of the interferometer due to the flat surface of He II. The resulting contrast depends also on the opened fraction *ν* of the grating, with *C* = 0.5 for *ν* = 0.45. It should be noted that since the source is mainly incoherent (*ℓ*_0_ ≈ *d*/4), the real advantage of the superthermal beam is that the acceptable high-contrast region is relatively wide (>1 μm) along the optical axis. This allows for grating misalignment (tip-tilt) in the longitudinal direction, leaving the rotational alignment of G2 and G3 the only critical point. On the basis of the small size (*L* ≈ 10 mm), monolithic Si devices are considered.

The above contrast calculation is conservative, as the effective aperture for this directed beam (*A*_eff_ < 1 mm, region of G1 from where particles can reach G3) is substantially smaller than the source size, which should account as further collimation. Another clear advantage is the nearly mono-energetic nature of the beam, which predicts a nearly uniform interaction time and phase shift under gravity.

Measuring a gravitational phase shift requires a horizontal beam geometry. A straightforward way is to direct the Mu atoms horizontally, using a mirror surface coated by the He II film. To avoid the lifetime losses associated with this, our ongoing developments concentrate on the direct synthesis of horizontal beams, using nanofabricated Si structures that can support He II layers in a vertical geometry by the capillary force.

### Comparison of various Mu beams for laser spectroscopy

To compare the suitability of different Mu sources for 1S–2S laser spectroscopy, we simulated Mu emission from three sources with their respective beam characteristics. The absolute vacuum Mu rate (Table 1) was calculated as *R*_Mu_ = *η*_Mu_ × *R*_μ_, where the conversion efficiency *η*_Mu_ depends on the μ^+^ momentum. To estimate *R*_μ_, representing the maximal deliverable μ^+^ rate to a 10 × 10-mm^2^ target, we considered two beamlines at PSI. For subsurface muons, the πE5 beamline delivers *R*_μ_ = 1.5 × 10^8^ μ^+^ s^−1^ at *p*_μ_ = 28 MeV *c*^−1^ to a *σ*_*x*_ ≈ *σ*_*y*_ ≈ 10 mm beam spot^61^. Here the muon rate follows a power law of ${R}_{\mu }\propto {p}_{\mu }^{3.5}$ (ref. ^62^). For low-energy muons, the LEM beamline delivers *R*_μ_ = 3 × 10^3^μ^+^ s^−1^ at ${E}_{\mu }^{{\rm{kin}}}$ = 5 keV (*p*_μ_ = 1 MeV *c*^−1^) to a *σ*_*x*_ ≈ *σ*_*y*_ ≈ 7 mm beam spot^63^. Note that Table 1 does not consider the variation in *R*_μ_, which, due to accelerator conditions, can be on the level of 10%.

To estimate the number of atoms addressable by a continuous-wave laser, we defined ${\phi }_{{\rm{Mu}}}^{{\rm{laser}}}$ as the flux of Mu atoms propagating through two 1 × 1-mm^2^ planes separated by 1 mm positioned at varying distances from the target surface. Compared with the proposed cryogenic SiO_2_ source, the He II source increases the number of atoms that the laser addresses by a factor of ~800.

## Online content

Any methods, additional references, Nature Portfolio reporting summaries, source data, extended data, supplementary information, acknowledgements, peer review information; details of author contributions and competing interests; and statements of data and code availability are available at https://doi.org/10.1038/s41567-026-03433-x.

## Supplementary information

Peer Review file

## Data Availability

The source data underlying Figs. 2 and and 3 and Extended Data Figs. 2–5 are available in the ETH research collection at https://doi.org/10.3929/ethz-c-000802445 (ref. ^64^).

## Code availability

The analysis code is available from the corresponding author upon reasonable request.
