# Supplementary material for: Generation of a high-intensity, superthermal muonium beam for gravity and laser spectroscopy experiments
Source: Nat Phys. 2026 Sep 14;22(11):1748–53. doi: 10.1038/s41567-026-03433-x (PMC13759314; doi:10.1038/s41567-026-03433-x)
Supplement: Supplementary file 1 [file 41567_2026_3433_MOESM1_ESM.pdf]

nature portfolio

Peer Review File

Generation of a high intensity, superthermal muonium beam for gravity and laser spectroscopy experiments

Corresponding Author: Professor Anna Soter

This manuscript has been previously reviewed at another journal. This document only contains information relating to versions considered at Nature Physics.

Version 0:

Reviewer comments:

Reviewer #1

(Remarks to the Author)
In the manuscript “Synthesis of a high intensity, superthermal muonium beam for gravity and laser spectroscopy experiments” by a group of physicists from Switzerland and Germany, the authors reached a new milestone in the technology of a high-brightness muonium production in vacuum by shooting the well-controlled accelerator muon beam at PSI towards a thin layer of superfluid helium (SFHe) with the high isotropic purity after various optimizations of the target properties. In history, numerous experiments explored the use of Silica powder and aerogel to form muoniums at the relatively low efficiency. Most of muoniums are stuck and absorbed in the target material while a few reaches the vacuum via thermal diffusion, causing broad energy and angular distributions. This drawback has hindered the progress of muonium physics for a while. Since the binding energy of muonium is much smaller than the chemical potential of SFHe, the novel method using SFHe will propel the formed muonium into vacuum as an almost sharp velocity by the Pauli repulsion. Due to the effects of gravity and surface tension, the thick SFHe target would suffer from beam-induced heating that disrupts the superfluid state and significantly reduces the muonium emission probability while the thin SFHe films might not be sufficient to effectively stop the incident muons. The current work showed the highly efficient production of the vacuum muonium yield with the SFHe target comparable to the best-performing room temperature. The breakthrough of the muonium beam in this study will pave the way for muonium interferometry experiments, like LEMING/MAGE to test the weak equivalence principle and the muonium 1S-2S laser spectroscopy to conduct precision measurement of QED as well as searching for new physics beyond standard model. Therefore, the results presented in this work are of great interests and broad importance to the muon/muonium physics community and certainly merits a publication in Nature Physics.
Authors are experienced and respected experimentalists with a long record of muon/muonium physics studies. The results obtained in the study are highly impressive and laid foundations for future muonium physics. The conclusions drawn in the manuscript are valid and well supported. The presentation is thorough, transparent and the discussion is sound and well justified. Data are well interpreted. I am convinced by the intelligent design of the experiments and enjoy the reading very much.
There are some issues which could be addressed prior to publication, particularly regarding the details and some of the outlooks made in the paper:
(1) I fully agree with authors that carrying out this experiment was inherently challenging due to the short lifetime of the muon+ and the difficulties to synthesize the muonium atom in vacuum and determine the production efficiency by the coincident detection system. The explanation of experimental data relied on several unknown factors and tuning parameters in the Monte Carlo (MC) simulation, such as the exact value of chemical potential, the muonium formation probability at $T<0.7$ K in SFHe, the muonium thermalization and diffusion in the target. For this reason and in the context of error propagations on these aspects, I would like to know more detailed description of the MC simulations. After all, we must see the uncertainties of the numerical results in Table I.
(2) One of the important directions to push forward the high muonium formation in vacuum is to search for new physics beyond standard model via a muonium to antimuonium conversion process or muonium rare decays. Suppose we in the distant future acquire much higher muonium beams for physics studies. This study also offers an avenue for high-yield vacuum muonium formation. I recommend the citation of the related conceptual design report for the next generation muonium-antimuonium conversion experiment (MACE) [Nucl. Sci. Tech. 37 (2026) 4, 57], though it remains unknown how to combine the current muonium source with the MACE detection system. This reference could follow in Line 40.
(3) Figure 1 is very presented and looks awesome. However, it still takes me some time to understand the real dimension in the 3D drawings. The scale bar 20 mm was put on the axes? I would expect a separate scale bar somewhere in the panel

(a). In the panel (c), what is LF, LC, RC and RF? I realized the meaning of different plastic scintillator detectors after a deep digestion at the section Method. Are they pointing to Left Far (LF), Left Close (LC), Right Close (RC) and Right Far (RF) detectors?
(4) In the caption of Fig.1, the average depth of SFHe seems like an assumption since authors put an “approximate” sign there. After reading the section Method, the data analyses could give a quantitative result on the statement. I would suggest a citation for the related section.
(5) In Line 96, the cited references [33, 32] seem awkward. There is a recent publication on the optimization of muonium yield in perforated silica aerogel [Phys. Rev. D 109 (2024) 7, 072012]. I recommend the relevant citation there.
(6) In Figure 2, The fit in the red line did not match the data very well. It seems that some oscillation features show up apart from the exponential term. Could you please explain the phenomenon?
(7) In the section Method, it seems missing of the vacuum level for the whole complex system. Could you please give us a hint?
(8) We identified the formation of muonium in vacuum after the collision of muon beams on the SFHe target by tracking the muonium decay productions (positrons) with the coincidence measurements. Since there positrons are charged particles, are there impacts from the EM field in the muon transportation beamline? I would expect some contributions in the data analyses.
(9) As authors told us that the isotopically pure He-4 played an important role in the results. The commercially available 6N Helium gas could not offer the good vacuum emission. Could you please tell us how to measure the purity of He-4? I suspect something like ICP-MS or how often in the data taking period?
(10) Line 559, there is a typo. “asses” should be “assess”?

Reviewer #2

(Remarks to the Author)
A. Summary

This report is of an important demonstration of the production of a high-brightness muonium beam in vacuum. This project brings together a number of disparate techniques from cryogenics, accelerator physics, etc to provide a convincing demonstration of the production of this novel beam. This technique will open new avenues for the performance of high precision studies in gravitation, exotic atom physics, and fundamental particle physics.

B. Originality and Significance

This work is certainly novel and signficant. As far as I am aware, the idea of applying the chemical potential driven expulsion of hydrogenic atoms in superfluid helium to the production of muonium beams belongs originally to one of the authors of the current paper (Takku, reference 37). The practical implementation of that idea is extremely technically challenging, requiring the production of novel beamline, cryogenic, and measurement apparatus. The muon beam manipulation in the momentum range needed for this project in particular is extremely challenging, requiring extremely tight tolerances and beamline control. Demonstration of this technique is provided by the convincing data in this paper. This demonstration will open up new avenues for other projects to utilize the same techniques for other areas of high interest in a variety of fields.

C. Data and Methodology

The data collected and the analysis methodology applied to them are sufficient to convince me that the paper's claims are accurate and will be reproducible by others who attempt to replicate the results.

D. Statistics and uncertainties

The statistical methods needed for the analyses here are not particularly sophisticated, and appear to have been applied consistent with best practices. The uncertainties also appear to have been handled appropriately, and the conclusions drawn are consistent with the calculated uncertainties.

E. Conclusions

The conclusions of the paper follow directly from the analysis presented: the team has succeeded in forming a superthermal, high-brightness muonium beam from positive muon stops in superfluid helium. This type of beam will lead to significant improvements in the sensitivities of a number of measurements, and will allow the measurement of a few novel phenomena.

F. Suggested improvements

I do not have any significant concerns about experimental methodology, and do not have any improvements to suggest. The suggestions I do have are minor and I would not be opposed to publication if the authors choose to decline to address them at this stage.

1. Motivation: The scientific motivation for the project, at least in this presentation, is highly weighted towards measuring the gravitation interactions of leptonic antimatter. In my discussions with colleagues, the gravitation angle was widely panned by the muon community as lacking in scientific motivation. The question (which was never in much doubt) is widely considered settled by the antihydrogen gravitational measurements, and the counterpoint in the present article is a fairly weak refutation. The spectroscopy and fundamental physics potential of this beam are significantly stronger. I have my own thoughts, but the

most compelling use to me would be a new, clean search for muonium-antimuonium CLFV oscillations. Again, I realize this is not the authors' interest, but these are more compelling motivations in the wider community.

2. Future directions

I would be very interested if the authors have more to say on the future potential for improving the intensity of their beam source, and the key challenges. The muonium beam they have reported on is already useful for a range of experiments, but improvements by a few orders of magnitude would be extremely interesting to a number of other potential projects in fundamental physics.

G. References

The selection of references is indeed appropriate to this paper. I did not notice any significant missing credits due to other authors.

H. Clarity and context

The results were placed well in the context of the field (modulo my comment above about the strength of the motivation). I was impressed with the clarity and lucidity of the presentation, which made the article a pleasure to read and learn from. There was one paragraph in the article (from lines 256 through 266 inclusive) that required a few reads to finally understand, but otherwise I have no issues.

Reviewer #3

(Remarks to the Author)
The LEMING experiment is a pioneering experiment at PSI designed to measure the gravitational acceleration of muonium, an "exotic atom" consisting of a positive muon (antimatter) and an electron, using atom interferometry. By studying whether antimatter/exotic systems fall at the same rate as normal matter, the experiment seeks to perform a direct test of Albert Einstein's Weak Equivalence Principle (WEP) with an antimatter-dominated, purely leptonic system, a fundamental requirement to search for physics beyond the Standard Model. If successful, the LEMING collaboration will provide one of the first direct measurements of the effect of gravity on a composite system dominated by antimatter and not composed of hadrons, as in the case of antihydrogen.

This paper describes the first milestone achieved by the collaboration in demonstrating the production of a novel cold muonium beam using superfluid helium. Because muonium has a short lifetime, a high-intensity, cold, and slow-moving beam is necessary to measure its free fall. LEMING utilizes a unique and clever method by stopping standard, high-energy muons in a thin layer of superfluid helium-4, which is used because it is inert and forces impurity atoms (like the newly formed muonium) out of its bulk, creating a high-brightness beam of cold muonium atoms that are emitted into a vacuum. This technique creates a non-thermal, slow beam with a narrow energy spread, which is essential for precise, low-energy measurements and achieving the LEMING final goal. The authors describe the corresponding experiments and the apparatus used very well and in sufficient detail. The analysis of the data is clear, and the Monte Carlo simulations support the findings. The team can be congratulated on such a nice experiment. The implications for a gravity experiment using this new cold muonium beam are relatively well described, accompanied by calculations, and promising.

These experimental results are also a real progress in the field of low-energy muon beams, which will have implications on many other precision muon experiments like the muonium 1S-2S laser spectroscopy discussed in the paper, but also possibly for the production of an improved low-energy muon beam for material science studies, and, after reacceleration, for realizing a muon microscope and the muon g-2 experiment at J-PARC.

Therefore, I consider the present paper interesting for a broad audience in physics. It is also well-written, and no mistakes whatsoever could be spotted. It is extensively referenced, giving appropriate credit to previous work, and its style seems to me to be entirely appropriate for publication in Nature Physics. I recommend the publication of this manuscript in Nature Physics, provided that the few comments below are addressed.

Comments:

(1) When describing the feasibility of a Mu gravity experiment (line 278, as well as line 575), please clarify how the vertical ascending superthermal beam presented here will be used for a gravity test where the tree-grating interferometer must be placed horizontally. It would be beneficial for the reader to add a brief description.

(2) Table 1: Please add the relevant references for the data relating to "Aerogel" and "SiO2 film", either in the table or in the caption. For clarity, please also indicate that "Aerogel" is at room temperature (300 K) and "SiO2 film" is at 100 K.

(3) line 108: define the acronym LEMING.

(4) line 344: define the acronym CHRISP.

(5) line 448: Fig. 9 comes before Fig. 7 and Fig. 8. Please rearrange the order.

(6) line 477: Fig. 2c does not exist. The authors may have meant to refer to Fig. 3c.

Version 1:

Reviewer comments:

Reviewer #1

(Remarks to the Author)
The authors have made revisions to resolve all my concerns and answered the critical questions.
I am happy to propose accepting the article for publication in Nature Physics.
Congratulations!

Reviewer #2

(Remarks to the Author)
The authors have addressed all of the comments from my initial review of the manuscript, and I have no additional comments or concerns. I appreciate the thoroughness of the responses to the comments and the changes to the manuscript. I would recommend publication of this important result.

Reviewer #3

(Remarks to the Author)
I confirm that the authors have adequately addressed the shortcomings of the original submission point by point and that these have been well incorporated into the revised manuscript. I also consider the key issues raised by the other reviewers to be relevant, and I believe the authors' responses to them are appropriate. With all these changes applied, the revised manuscript is now of very high quality, and I am pleased to recommend it for publication in Nature Physics.

Open Access This Peer Review File is licensed under a Creative Commons Attribution 4.0 International License, which permits use, sharing, adaptation, distribution and reproduction in any medium or format, as long as you give appropriate credit to the original author(s) and the source, provide a link to the Creative Commons license, and indicate if changes were made.
In cases where reviewers are anonymous, credit should be given to 'Anonymous Referee' and the source.
The images or other third party material in this Peer Review File are included in the article’s Creative Commons license, unless indicated otherwise in a credit line to the material. If material is not included in the article’s Creative Commons license and your intended use is not permitted by statutory regulation or exceeds the permitted use, you will need to obtain permission directly from the copyright holder.
To view a copy of this license, visit https://creativecommons.org/licenses/by/4.0/

Manuscript ID: NPHYS-2026-01-00347-T
Title: Synthesis of a high intensity, superthermal muonium beam for gravity and laser spectroscopy experiments
Corresponding author: Prof. Anna Soter

RESPONSE TO REVIEWERS

We thank the reviewers for their thoughtful and constructive feedback on our manuscript. We have addressed all the questions raised in sequence below, and we believe the manuscript is significantly strengthened as a result. Below is our point-by-point response to the reviewers' comments.

Comments of the reviewers are italic.
The author's response is in blue.
Changes in the paper are highlighted in teal.

REVIEWER #1

In the manuscript "Synthesis of a high intensity, superthermal muonium beam for gravity and laser spectroscopy experiments" by a group of physicists from Switzerland and Germany, the authors reached a new milestone in the technology of a high-brightness muonium production in vacuum by shooting the well-controlled accelerator muon beam at PSI towards a thin layer of superfluid helium (SFHe) with the high isotropic purity after various optimizations of the target properties. In history, numerous experiments explored the use of Silica powder and aerogel to form muoniums at the relatively low efficiency. Most of muoniums are stuck and absorbed in the target material while a few reaches the vacuum via thermal diffusion, causing broad energy and angular distributions. This drawback has hindered the progress of muonium physics for a while. Since the binding energy of muonium is much smaller than the chemical potential of SFHe, the novel method using SFHe will propel the formed muonium into vacuum as an almost sharp velocity by the Pauli repulsion. Due to the effects of gravity and surface tension, the thick SFHe target would suffer from beam-induced heating that disrupts the superfluid state and significantly reduces the muonium emission probability while the thin SFHe films might not be sufficient to effectively stop the incident muons. The current work showed the highly efficient production of the vacuum muonium yield with the SFHe target comparable to the best-performing room temperature. The breakthrough of the muonium beam in this study will pave the way for muonium interferometry experiments, like LEMING/MAGE to test the weak equivalence principle and the muonium 1S-2S laser spectroscopy to conduct precision measurement of QED as well as searching for new physics beyond standard model. Therefore, the results presented in this work are of great interests and broad importance to the muon/muonium physics community and certainly merits a publication in Nature Physics. Authors are experienced and respected experimentalists with a long record of muon/muonium physics studies. The results obtained in the study are highly impressive and laid foundations for future muonium physics. The conclusions drawn in the manuscript are valid and well supported. The presentation is thorough, transparent and the discussion is sound and well justified. Data are well interpreted. I am convinced by the intelligent design of the experiments and enjoy the reading very much. There are some issues which could be addressed prior to publication, particularly regarding the details and some of the outlooks made in the paper:

We would like to thank the Reviewer for this detailed feedback. We appreciate the acknowledgment of our physics motivations and results, and the insightful comments.

(1) I fully agree with authors that carrying out this experiment was inherently challenging due to the short lifetime of the muon+ and the difficulties to synthesize the muonium atom in vacuum and determine the production efficiency by the coincident detection system. The explanation of experimental data relied on several unknown factors and tuning parameters in the Monte Carlo (MC) simulation, such as the exact value of chemical potential, the muonium formation probability at T<0.7 K in SFHe, the muonium thermalization and diffusion in the target. For this reason and in the context of error propagations on these aspects, I would like to know more detailed description of the MC simulations. After all, we must see the uncertainties of the numerical results in Table I.

We extended the description of the MC simulations in Methods, particularly with details on how the conversion efficiency η_{Mu} and its uncertainty as given in Table I are obtained (lines 573-583) using parameter scans and direct comparison of the MC result and the measured time histograms. The uncertainties of the projected muonium rates in Table I (R_{Mu}) were propagated purely from this η_{Mu}, which was obtained for the superthermal source in the present work, while η_{Mu} of the best performing thermal sources was taken from the cited literature.

Since these three sources require different muon beam momenta, we used the nominal momentum-dependent muon rates ($R_{\mu}(p_{\mu})$) at the highest intensity (sub)surface muon beamline (πE5) and ultralow energy LEM beamline at PSI to give a fair comparison. The muon rates are used here as scaling factors without uncertainties. These values also stand for the globally highest available continuous (cw) muon rates at the given momentum.

We would also point out that the main characteristics of the new source (conversion efficiency, diffusion and propagation velocities) were also estimated independently of the MC simulations, as described briefly in the main text (lines 193-199), and in detailed in Methods (lines 478-494). From direct counts of the number of escaping Mu atoms per stopped muons in the detectors (lines 499-505) we estimate the emission efficiency to be $\eta_{\mathrm{Mu}} = (8.6 \pm 0.8)\%$ in agreement with the results obtained from the full MC parameter scans, the latter simply providing a more conservative estimation to the uncertainties.

(2) One of the important directions to push forward the high muonium formation in vacuum is to search for new physics beyond standard model via a muonium to antimuonium conversion process or muonium rare decays. Suppose we in the distant future acquire much higher muonium beams for physics studies. This study also offers an avenue for high-yield vacuum muonium formation. I recommend the citation of the related conceptual design report for the next generation muonium-antimuonium conversion experiment (MACE) [Nucl. Sci. Tech. 37 (2026) 4, 57], though it remains unknown how to combine the current muonium source with the MACE detection system. This reference could follow in Line 40.

We thank for the helpful comment and suggestion. We cite the existing limit on Mu-anti-Mu conversion in our introduction (Ref. [9]), and the MACE experiment among future motivations (Ref. [12]). While the advantages of this high quality cryogenic source is clear for spectroscopy and interferometry, the application for such conversion experiments needs further investigation. We extended our concluding lines in the light of this (line 343).

(3) Figure 1 is very presented and looks awesome. However, it still takes me some time to understand the real dimension in the 3D drawings. The scale bar 20 mm was put on the axes? I would expect a separate scale bar somewhere in the panel (a). In the panel (c), what is LF, LC, RC and RF? I realized the meaning of different plastic scintillator detectors after a deep digestion at the section Method. Are they pointing to Left Far (LF), Left Close (LC), Right Close (RC) and Right Far (RF) detectors?

For less ambiguity, we removed the scale from Figure 1 where the 3D interpretation is hard to perceive, and implemented it in panel (c) only, where the scale is relevant. We also provided an explicit description of the naming of detector channels in the caption for a better read.

(4) In the caption of Fig.1, the average depth of SFHe seems like an assumption since authors put an "approximate" sign there. After reading the section Method, the data analyses could give a quantitative result on the statement. I would suggest a citation for the related section.

We thank for this suggestion - we direct the reader in the main text to Methods (lines 381-400) where we describe the corresponding simulation in detail, and removed the ambiguous sign.

(5) In Line 96, the cited references [33, 32] seem awkward. There is a recent publication on the optimization of muonium yield in perforated silica aerogel [Phys. Rev. D 109 (2024) 7, 072012]. I recommend the relevant citation there.

We have initially cited this work at a different location but agree with Reviewer 1 that it is also suitable at this location. We have thus adapted the references.

(6) In Figure 2, The fit in the red line did not match the data very well. It seems that some oscillation features show up apart from the exponential term. Could you please explain the phenomenon?

Oscillations could originate from muonium spin precession on the ~ 500 ns timescale in the residual fringe magnetic fields and the Earth magnetic field. We (mainly) cancel them by summing the counts of left and right-sided detectors. While these residuals might be still visible, we attribute the features at late times to statistical fluctuation (due to lifetime losses, the available muons exponentially decrease, which is also reflected by the increasing uncertainties on the bins).

We would also note that the above fits of a generic skewed distribution are only used to obtain the peak position and hence a model-independent estimation on the propagation velocity, (and to provide some reasonable parameter range for the Monte Carlo analysis).

(7) In the section Method, it seems missing of the vacuum level for the whole complex system. Could you please give us a hint?
The pressure in the muon beamline and the outer vacuum can is $p \approx 5 \times 10^{-7}$ mbar. The vacuum inside the target chamber is defined by the saturated vapor pressure of He, which at $T = 0.2$K is expected to be $p \approx 10^{-15}$ mbar. This information has been added (lines 379-383).

(8) We identified the formation of muonium in vacuum after the collision of muon beams on the SFHe target by tracking the muonium decay productions (positrons) with the coincidence measurements. Since there positrons are charged particles, are there impacts from the EM field in the muon transportation beamline? I would expect some contributions in the data analyses.
The strength of the fringe fields of the closest beamline element at the location of the target chamber is in the order of µT. The Michel positrons from the muon decay are energetic (the majority in between $\approx 20 - 50$ MeV, close to minimum ionizing), so we can safely neglect the effect of the beamline elements on the trajectories of the positrons. We would like to add that we have studied in detail the more important concern, namely the scattering of the positrons on the 1 mm Cu wall of the target chamber - but found no impact on our detector resolution.

(9) As authors told us that the isotopically pure He-4 played an important role in the results. The commercially available 6N Helium gas could not offer the good vacuum emission. Could you please tell us how to measure the purity of He-4? I suspect something like ICP-MS or how often in the data taking period?
Isotropic purification can be done by an engineered superleak and a cryostat, but it is tricky at this level of purity ($\sim 10^{-12}$). Hence we obtained the gas from our colleagues in Lancaster (in Acknowledgements). They produce this record purity isotopic gas commercially, mainly for ultracold neutron experiments - and indeed mass spectrometry was used to verify the purity before the measurement. During measurement, we simply took special care to keep our gas system clean with UHV components (VCR and welded connections, TM pumps) and strict gas flushing (cleaning cycles) with the pure gas.

(10) Line 559, there is a typo. "asses" should be "assess"?
Indeed, an annoying error that the spell checker ignored - we have corrected this!

REVIEWER #2

A. Summary

This report is of an important demonstration of the production of a high-brightness muonium beam in vacuum. This project brings together a number of disparate techniques from cryogenics, accelerator physics, etc to provide a convincing demonstration of the production of this novel beam. This technique will open new avenues for the performance of high precision studies in gravitation, exotic atom physics, and fundamental particle physics.

B. Originality and Significance

This work is certainly novel and significant. As far as I am aware, the idea of applying the chemical potential driven expulsion of hydrogenic atoms in superfluid helium to the production of muonium beams belongs originally to one of the authors of the current paper (Takku, reference 37). The practical implementation of that idea is extremely technically challenging, requiring the production of novel beamline, cryogenic, and measurement apparatus. The muon beam manipulation in the momentum range needed for this project in

particular is extremely challenging, requiring extremely tight tolerances and beamline control. Demonstration of this technique is provided by the convincing data in this paper. This demonstration will open up new avenues for other projects to utilize the same techniques for other areas of high interest in a variety of fields.

C. Data and Methodology

The data collected and the analysis methodology applied to them are sufficient to convince me that the paper's claims are accurate and will be reproducible by others who attempt to replicate the results.

D. Statistics and uncertainties

The statistical methods needed for the analyses here are not particularly sophisticated, and appear to have been applied consistent with best practices. The uncertainties also appear to have been handled appropriately, and the conclusions drawn are consistent with the calculated uncertainties.

E. Conclusions

The conclusions of the paper follow directly from the analysis presented: the team has succeeded in forming a superthermal, high-brightness muonium beam from positive muon stops in superfluid helium. This type of beam will lead to significant improvements in the sensitivities of a number of measurements, and will allow the measurement of a few novel phenomena.

We thank the Reviewer for this careful assessment, and the positive feedback on the presented novel muonium source, especially concerning the recognition of its impact.

F. Suggested improvements

I do not have any significant concerns about experimental methodology, and do not have any improvements to suggest. The suggestions I do have are minor and I would not be opposed to publication if the authors choose to decline to address them at this stage.

1. Motivation: The scientific motivation for the project, at least in this presentation, is highly weighted towards measuring the gravitation interactions of leptonic antimatter. In my discussions with colleagues, the gravitation angle was widely panned by the muon community as lacking in scientific motivation. The question (which was never in much doubt) is widely considered settled by the antihydrogen gravitational measurements, and the counterpoint in the present article is a fairly weak refutation. The spectroscopy and fundamental physics potential of this beam are significantly stronger.

We agree that the prospect for sub-kHz laser spectroscopy of the 1S-2S transition is extremely promising, and has various ways to impact beyond SM searches as well (see eg. [17]). With our roots in precision spectroscopy, we naturally plan to pursue this experiment as one of our important scientific goals.

As of the gravity experiment: we are rather convinced that this measurement is complementary to studies using antihydrogen - and based on frequent discussions with the antiproton community, we seem to agree on this issue. We would like to highlight that our main interest is not to compare matter with antimatter, but:

(1) to directly test the weak equivalence principle for the first time using second generation matter (the muon, which happens to be the antimuon). We are hence probing whether there is a flavour-dependent coupling of gravity.

(2) to carry out a 'clean' measurement of gravitational coupling to elementary fermion masses. In muonium, the elementary muon and electron masses attributed to the Higgs mechanism determine the first $\sim$7 digits of the atomic mass. In the composite hadrons (proton, neutron and antiproton, in fact all matter we have seen gravitating), most of the mass is generated by the complex relativistic dynamics during strong confinement in a model-dependent way. This question - what do we actually compare when we drop an antiproton vs the proton - was frequently raised concerning antihydrogen gravity experiments, and could be answered by leptons.

(3) even in the absence of any exotic gravity, the free fall of muonium in the gravitational field of Earth (which is full of electrons) provides a sensitive probe to flavour-dependent new interactions already at the 10% precision, as its showed by Stadnik [15]. Kostelecky's more generic effective field theory framework [17] also allows for flavour-dependent anomalies. We plan to explore the parameter space and put new limits on such new interactions, together with 1S-2S spectroscopy.

To make the above arguments more clear, we slightly reformulated the corresponding part in the main text (Lines 70-77).

I have my own thoughts, but the most compelling use to me would be a new, clean search for muonium-antimuonium CLFV oscillations. Again, I realize this is not the authors' interest, but these are more compelling motivations in the wider community.

The muonium-antimuonium CLFV oscillations (cited in Refs [9,12]) also comprise an interesting subject, and we thank the Reviewer for this mention. While its clear how laser spectroscopy and interferometry benefits from this new source, it needs further investigation if there is a real benefit to use it in muonium-antimuonium conversion experiments. We added a mention (line 343) in the conclusions of the main text.

2. Future directions

I would be very interested if the authors have more to say on the future potential for improving the intensity of their beam source, and the key challenges. The muonium beam they have reported on is already useful for a range of experiments, but improvements by a few orders of magnitude would be extremely interesting to a number of other potential projects in fundamental physics.

The relatively high conversion efficiency ($\eta_{\mathrm{Mu}} = (8.2^{+0.8}_{-1.1})$, stopped muon to vacuum muonium) shows that the converter itself cannot be improved by orders of magnitude, put percentile level changes can be expected by optimizing particle stopping and different energies, which will follow in a separate study.

Besides this, we expect up to two orders of magnitude more surface muon (and hence muonium) rates by using the next generation production target (HIMB beamlines), which is under development at PSI. We put a mention of this in the text (line 308-310).

G. References

The selection of references is indeed appropriate to this paper. I did not notice any significant missing credits due to other authors.

H. Clarity and context

The results were placed well in the context of the field (modulo my comment above about the strength of the motivation). I was impressed with the clarity and lucidity of the presentation, which made the article a pleasure to read and learn from. There was one paragraph in the article (from lines 256 through 266 inclusive) that required a few reads to finally understand, but otherwise I have no issues.

We modified this paragraph to improve clarity (lines 260-268), and added further guidance in the captions of Figure 2 and 3.

REVIEWER #3

The LEMING experiment is a pioneering experiment at PSI designed to measure the gravitational acceleration of muonium, an "exotic atom" consisting of a positive muon (antimatter) and an electron, using atom interferometry. By studying whether antimatter/exotic systems fall at the same rate as normal matter, the experiment seeks to perform a direct test of Albert Einstein's Weak Equivalence Principle (WEP) with an antimatter-dominated, purely leptonic system, a fundamental requirement to search for physics beyond the Standard Model. If successful, the LEMING collaboration will provide one of the first direct measurements of the effect of gravity on a composite system dominated by antimatter and not composed of hadrons, as in the case of antihydrogen.

This paper describes the first milestone achieved by the collaboration in demonstrating the production of a novel cold muonium beam using superfluid helium. Because muonium has a short lifetime, a high-intensity, cold, and slow-moving beam is necessary to measure its free fall. LEMING utilizes a unique and clever method by stopping standard, high-energy muons in a thin layer of superfluid helium-4, which is used because it is inert and forces impurity atoms (like the newly formed muonium) out of its bulk, creating a high-brightness beam of cold muonium atoms that are emitted into a vacuum. This technique creates a non-thermal, slow beam with a narrow energy spread, which is essential for precise, low-energy measurements and achieving the LEMING final goal. The authors describe the corresponding experiments and the apparatus used very well and in sufficient detail. The analysis of the data is clear, and the Monte Carlo simulations support the findings. The team can be congratulated on such a nice experiment. The implications for a gravity experiment using this new cold muonium beam are relatively well described, accompanied by calculations, and promising.

These experimental results are also a real progress in the field of low-energy muon beams, which will have implications on many other precision muon experiments like the muonium 1S-2S laser spectroscopy discussed in the paper, but also possibly for the production of an improved low-energy muon beam for material science studies, and, after reacceleration, for realizing a muon microscope and the muon g-2 experiment at J-PARC.

Therefore, I consider the present paper interesting for a broad audience in physics. It is also well-written, and no mistakes whatsoever could be spotted. It is extensively referenced, giving appropriate credit to previous work, and its style seems to me to be entirely appropriate for publication in Nature Physics. I recommend the publication of this manuscript in Nature Physics, provided that the few comments below are addressed.

We thank the Referee for the encouraging statements concerning the presented results, and for the useful suggestions.

Comments:

When describing the feasibility of a Mu gravity experiment (line 278, as well as line 575), please clarify how the vertical ascending superthermal beam presented here will be used for a gravity test where the tree-grating interferometer must be placed horizontally. It would be beneficial for the reader to add a brief description.

It is indeed true, we need a the same beam propagating horizontally for a gravity measurement. The obvious option with the present setup would be to utilize an optically polished surface (a mirror) coated with superfluid helium film for directing the atoms. However, this solution adds further lifetime losses.

To avoid this, our group just recently developed vertical microfabricated structures that collect a layer of superfluid helium directly behind the first grating, and have observed horizontal vacuum Mu emission from such structures. We are currently optimizing this new geometry and quantifying the quality of the horizontal beam for a separate study. To clarify efforts in this direction, we have added short mentions to the main text (line 305, 348) and some explanations in Methods (lines 657-664).

Table 1: Please add the relevant references for the data relating to "Aerogel" and "SiO2 film", either in the table or in the caption. For clarity, please also indicate that "Aerogel" is at room temperature (300 K) and "SiO2 film" is at 100 K.

We have added the temperature as well as the corresponding reference.

line 108: define the acronym LEMING.
line 344: define the acronym CHRISP.

The acronyms have been defined in the adapted manuscript.

line 448: Fig. 9 comes before Fig. 7 and Fig. 8. Please rearrange the order.

Thank you for spotting this issue, we adapted the order.

line 477: Fig. 2c does not exist. The authors may have meant to refer to Fig. 3c.

Thank you for this careful read and the observation. We corrected the error.
